# *Piriformospora indica* Reprograms Gene Expression in Arabidopsis Phosphate Metabolism Mutants But Does Not Compensate for Phosphate Limitation

**DOI:** 10.3389/fmicb.2017.01262

**Published:** 2017-07-12

**Authors:** Madhunita Bakshi, Irena Sherameti, Doreen Meichsner, Johannes Thürich, Ajit Varma, Atul K. Johri, Kai-Wun Yeh, Ralf Oelmüller

**Affiliations:** ^1^Institute of General Botany and Plant Physiology, Friedrich-Schiller-University Jena Jena, Germany; ^2^Amity Institute of Microbial Technology, Amity University Noida, India; ^3^School of Life Sciences, Jawaharlal Nehru University New Delhi, India; ^4^Institute of Plant Biology, Taiwan National University Taipei, Taiwan

**Keywords:** root expression profiles, PHT1, WRKY6, *Piriformospora indica*, phosphate starvation

## Abstract

*Piriformospora indica* is an endophytic fungus of Sebacinaceae which colonizes the roots of many plant species and confers benefits to the hosts. We demonstrate that approximately 75% of the genes, which respond to *P. indica* in Arabidopsis roots, differ among seedlings grown on normal phosphate (Pi) or Pi limitation conditions, and among wild-type and the *wrky6* mutant impaired in the regulation of the Pi metabolism. Mapman analyses suggest that the fungus activates different signaling, transport, metabolic and developmental programs in the roots of wild-type and *wrky6* seedlings under normal and low Pi conditions. Under low Pi, *P. indica* promotes growth and Pi uptake of wild-type seedlings, and the stimulatory effects are identical for mutants impaired in the PHOSPHATE TRANSPORTERS1;1, -1;2 and -1;4. The data suggest that the fungus does not stimulate Pi uptake, but adapts the expression profiles to Pi limitation in Pi metabolism mutants.

## Introduction

*Piriformospora indica*, an endophytic fungus of Sebacinaceae, colonizes the roots of many plant species and promotes their growth and performance (Peškan-Berghöfer et al., 2004; Waller et al., 2005, 2008; Shahollari et al., 2007; Oelmüller et al., 2009; Camehl et al., 2010; Nongbri et al., 2012; Varma et al., 2012; Jogawat et al., 2013; Ye et al., 2014). The fungus improves nutrition uptake from the soil to the host roots (Sherameti et al., 2005; Shahollari et al., 2005; Kumar et al., 2011) suggesting a strong fungal influence on the plant transport processes and metabolism. Mycorrhizal and beneficial root-colonizing fungi deliver phosphate (Pi) to the roots. Pi is taken up by the plants through Pi transporters, and low- and high-affinity Pi transporters of the PHOSPHATE TRANSPORTER1 (PHT1) family have been reported to be involved in the mycorrhizal pathway (Harrison et al., 2002; Garcia et al., 2016). While mycorrhizal plants contain fungus-inducible *PHT1* genes (Bucher, 2007; Chen et al., 2007; Walder et al., 2015), plants which do not interact with mycorrhizal fungi, such as Arabidopsis, lack inducible *PHT1* genes. The expression of their Pi transporter genes is independent of root colonization by endophytic root-colonizing microbes, but some members of the *PHT1* gene family respond to Pi deficiency (e.g., Chiou et al., 2001; Ai et al., 2009; Ayadi et al., 2015). In return, up to 50% of the carbon fixed by photosynthesis can be delivered to mycorrhizal fungi associated with the roots in natural ecosystems (Nehls et al., 2010).

Root colonization and nutrient exchange by *P. indica* results in substantial alterations in the root architecture, an effect which is highly host specific (cf. Lee et al., 2011; Dong et al., 2013). In Arabidopsis, *P. indica* stimulates root growth: while the primary root length is slightly reduced, lateral root growth and root hair development is promoted (e.g., Varma et al., 1999; Barazani et al., 2005; Vadassery et al., 2008, 2009a; Johnson and Oelmüller, 2009; Fakhro et al., 2010; Das et al., 2012, 2014; Lahrmann and Zuccaro, 2012; Lahrmann et al., 2013; Prasad et al., 2013; Venus and Oelmüller, 2013; Bakshi et al., 2015; Vahabi et al., 2015). Secondary metabolites, such as indole-3-acetaldoxime derivatives, hormones, and different defense compounds/pathways control the colonization of roots during Arabidopsis/*P. indica* interaction and thus influence root development (Sherameti et al., 2008; Camehl et al., 2011; Hilbert et al., 2012; Nongbri et al., 2012; Bakshi et al., 2015; Matsuo et al., 2015). *P. indica* also protects the roots by stimulating the antioxidant system under stress, which again influences developmental programs in response to environmental cues and the redox state of the roots (Baltruschat et al., 2008; Vadassery et al., 2009b; Harrach et al., 2013). To what extend root developmental and genetic programs are altered by endophytes such as *P. indica* is not completely understood. The quite different root responses of various plant species to *P. indica* demonstrate the important role of the host genetic programs (discussed in Lee et al., 2011; Dong et al., 2013).

Here, we investigate the role of Pi availability and the Arabidopsis transcription factor WRKY6 involved in the regulation of the plant Pi metabolism in the *P. indica*/Arabidopsis symbiosis. The Arabidopsis mutant lacking the WRKY6 transcription factor shows an altered response to LP stress (Chen et al., 2009; Bakshi et al., 2015), since WRKY6 is involved in regulating *PHOSPHATE1* (*PHO1*) expression. PHO1 is a Pi exporter and required for the transfer of Pi from root epidermal and cortical cells to the xylem. The *pho1* mutant has low shoot Pi and shows Pi deficiency symptoms, including poor shoot growth and overexpression of numerous Pi-deficiency responsive genes (cf. Wege et al., 2016). LP treatment reduced WRKY6 binding to the *PHO1* promoter and thus allows *PHO1* expression (Chen et al., 2009). Therefore, deletion of WRKY6 alters the root development of WT seedlings in a Pi-dependent manner (Bakshi et al., 2015). We compared the expression profiles of WT and *wrky6* seedlings grown under normal Pi (NP) and low Pi (LP) conditions and found that the response to *P. indica* is quite different in the roots of WT and *wrky6* seedlings, both under NP and LP conditions. This suggests that the genetic response of Arabidopsis to *P. indica* is highly dependent on the nutrient availability and genotype of the host. We also investigated the role of Arabidopsis Pi transporters in the symbiosis and demonstrate that Pi limitation, due to inactivation of the plant Pi transporters, cannot be compensated by *P. indica* colonization.

## Materials and Methods

### Growth Conditions of Plant and Fungus

WT, *wrky6*, *pht1;1* (At5g43350), *pht1;2* (At5g43370), and *pht1;4* (At2g38940) seeds and seeds of the *pht1;1 pht1;4* double knock-out line were surface sterilized and placed on Petri dishes containing MS (Murashige and Skoog, 1962) nutrient medium (with 13,7 g/l sucrose). After cold treatment for 48 h at 4°C, the plates were incubated for 10 days at 22°C under continuous illumination (100 μmol m^-2^ s^-1^). *P. indica* was cultured as described previously on Aspergillus minimal medium (Johnson et al., 2011). After 10 days of growth, all seedlings were either transferred to fresh plates (MS or plant nutrient medium (PNM) as indicated in the figure legends, or to soil, cf. **Figure 6**) for the different treatments. The procedure has been described in details in Johnson et al. (2011)^1^. For the arsenate assay, they were transferred to fresh MS medium containing 1 mM Pi (KH_2_PO_4_) and 200 μM sodium arsenate (V) for 3 weeks (cf. **Table 2**). Alternatively, they were transferred to PNM medium (without sucrose) for 2 weeks. The PNM medium contained a fungal *P. indica* plaque in the middle (co-cultivation with *P. indica*) or an agar plaque without fungal hyphae (control, without *P. indica*), as described previously (Vadassery et al., 2009a). The fungus was allowed to grow on the PNM medium for 1 week before the seedlings were transferred to the fungal lawn. Control plates were treated in the same way. To test whether simultaneous inactivation of *PHT1;1* and *PHT1;4* has a long-term effect on plant performance, the 10-day old seedlings were transferred from MS plants (cf. above) to soil and kept in a greenhouse for 4 weeks. For the plate experiments shown in **Figure 6** (TOP), the seedlings were transferred to PNM medium with NP for 3 weeks.

### Generation of the Homozygous Knock-Out Lines

The following SALK insertion lines were used for the *PHT1* genes: *pht1;1* (At5g43350; SALK_088586C/N666665), *pht1;2* (At5g43370; SALK_110194) and *pht1;4* (At2g38940; SALK_103881). Homozygocity was tested with gene-specific primer pairs (cf. Supplementary Table S1) and primers given on the SALK homepage^2^. The *pht1;1 pht1;4* double knock-out line was generated by crossing the two homozygote single knock-out lines. After confirmation of homozygocity of the two inactivated genes, the lack of PHT transporters was also confirmed with the arsenate resistance assay. The *wrky6* line was described in Bakshi et al. (2015).

### Arsenate Resistance Assay

Seeds were germinated on full MS medium. After 10 days they were transferred to MS medium with 1 mM Pi (KH_2_PO_4_) and 200 μM sodium arsenate (V) for 3 weeks. Growth occurred in continuous white light (80 μmol m^-2^ s^-1^).

### Co-cultivation Experiments

Co-cultivation of *A. thaliana* (WT, *wrky6*, as well as *pht1* single and double knock-out lines) with *P. indica* was performed under *in vitro* culture conditions on a nylon membrane placed on top of solidified PNM media (Johnson et al., 2011). For Pi stress treatment PNM media with two different Pi concentrations [2.5 mM (NP, control) and 0.25 mM (LP, Pi stress)] were used. For expression profiling, square Petri dishes were divided into two equal parts. On one plate two *P. indica* disks on Aspergillus medium, one in each part, and on another plate two disks without the fungus, were placed on each part and kept for 7 days. The disks were used for mock treatment. After 48 h of cold treatment and 10 days of growth as described above on MS medium, seedlings of equal sizes were used for the co-cultivation assays or mock treatment, using the pre-prepared plates. For each Pi concentration, 4 treatments were compared: WT, WT + *P. indica*, *wrky6* and *wrky6* + *P. indica*. Seedlings were maintained under two different Pi concentrations with and without *P. indica* as mentioned above for 3 days at 22°C and 70–80% humidity in a 16-h light/8-h dark cycle. Roots were harvested and frozen in liquid nitrogen for total RNA extraction. They were used for gene expression analyses.

For analysis of the *pht1* mutants, the same treatment was performed except that the seedlings were maintained in the two different Pi concentrations in normal Petri dishes for 14 days, before harvest for further analysis.

### Microarray Analyses

Total RNA from roots of colonized/uncolonized WT and *wrky6* mutants from three independent biological experiments grown under NP and LP conditions were harvested 3 days after transfer to the fresh plates. RNA from roots of mock-treated WT and *wrky6* mutants (agar plaques instead of *P. indica* plaques) were used as control. The 3-day time point was chosen because in preliminary experiments, we observed a strong regulation of a selected number of genes. For each treatment, identical amounts of RNA from three independent biological replicates were labeled and hybridized according to Agilent’s One-Color Microarray-Based Gene Expression Analysis (OAK Lab GmBH, Hennigdorf, Germany). Quality of RNA samples were checked by photometrical measurements with the Nanodrop 2000 spectrophotometer (Thermo Scientific) and then analyzed on agarose gels (2%) as well as by using the 2100 Bioanalyser (Agilent Technologies, CA, United States) for determining the RNA integrity and the exclusion of potential contaminants. After verifying the quality of RNA, the Low Input Quick Amp Labeling Kit (Agilent Technologies) was used for generation of fluorescent complementary RNA (cRNA). Default cRNAs were amplified by using oligo-dT primers labeled with cyanine 3-CTP (Cye-3) according to the manufacturer’s protocol. Cye-3-labeled probes were hybridized to 8 ×60 k custom-designed Agilent microarray chips. For hybridization, the Agilent Gene Expression Hybridization Kit (Agilent Technologies) was used. The hybridized slides were washed and scanned using the SureScan Microarray Scanner (Agilent Technologies) at a resolution of 3 micron generating a 20 bit TIFF file, respectively.

### Microarray Data Analysis

Data extractions from Images were performed using the Agilent’s Feature Extraction software version 11. Feature extracted data were analyzed using the DirectArray Version 2.1 software from Agilent. Normalization of the data was performed with DirectArray using the ranked median quantiles according to Bolstad et al. (2003). To identify significantly differentially expressed genes log_2_-fold changes are calculated and Student’s *t*-test was performed. In summary, raw data were normalized by rank median quantiles, intensity values from replicate probes were averaged, log_2_-ratios between the treatments were calculated and Student’s *t*-statistics applied to test for significance. Genes with log_2_-fold change <–1 or >1 and *p*-value < 0.05 were considered to be significantly different. All data show expression levels of genes regulated by *P. indica* relative to the control levels without *P. indica*. Differentially expressed genes were then assigned using the A. *thaliana* Gene Ontology software (TAIR’s GO annotations) (Berardini et al., 2004) and transcript abundance were classified based on their functional categories and pathways using the MapMan^3^ software.

The microarray data have been submitted to NCBI (GEO) under the accession number GSE63500.

### Real Time PCR Analyses

RNA was isolated from root tissues of WT and mutant seedlings at the time points indicated in the Sections “Result” and “Figure Legends” using the RNeasy Plant Mini Kit (Qiagen), and reverse-transcribed for quantitative real-time PCR (qRT- PCR) analyses, using an iCycler iQ real-time PCR detection system and iCycler software (version 2.2; Bio-Rad). cDNA was synthesized using the Omniscript cDNA synthesis kit (Qiagen, Hilden, Germany) with 1 μg of RNA. For the amplification of the reverse-transcription PCR products, iQ SYBR Green Supermix (Bio-Rad) was used according to the manufacturer’s protocol in a final volume of 20 μl. The iCycler was programmed to 95°C for 3 min; 40 x (95°C 30 sec, 57°C 15 s, 72°C 30 sec), 72°C for 10 min, followed by a melting curve program from 55°C to 95°C in increasing steps of 0.5°C. All reactions were performed from three biological and three technical replicates. The mRNA levels for each cDNA probe were normalized with respect to the plant *ACTIN2* mRNA level. Fold-induction values of target genes were calculated with the ΔΔCP equation of Pfaffl (2001) and related to the mRNA level of target genes as indicated in the Result section. Primer pairs used in this study are given in Supplementary Table S2. They were designed using the CLC Main Workbench program^4^.

### Pi Content Analysis

For Pi content analyses, the samples were dried in an oven at 105°C overnight. The samples were mixed with 2 ml of 65% HNO_3_ and kept for one hour at 160°C. The final volume was adjusted to 10 ml and the pH to 3.0–4.0. Finally, samples were mixed with ascorbic acid reagent and ammonium molybdate reagent (DIN 38405) and the Pi content was analyzed by the phosphomolybdenum blue reaction using the UV-160A spectrophotometer. Total Pi concentration was determined for the complete seedlings and expressed in nmol/g dry weight. Experiments were repeated three times with different biological replicas.

### Chlorophyll Fluorescence Measurements

Plant performance was measured for WT and the *pht1;1 pht1;4* double knock-out line using chlorophyll fluorescence measurements. After germination and 10 days on MS medium (cf. above), the seedlings were transferred to fresh PNM plates with LP or NP concentrations under high light intensity (300 μmol m^-2^ s^-1^) for 1 week. This high light intensity confers stress to the seedlings. The efficiency of the photosynthetic electron transfer describing the fitness of the plants was measured as Fv/Fm described by Maxwell and Johnson (2000) after dark adaptation of the seedlings for 20 min. The fluorescence parameters were measured with a FluorCam 700MF instrument and analyzed with the Flucam 5.0 software. The data are averages for 30 seedlings and three independent biological experiments.

## Results

### *P. indica* Regulates Different Genes in WT and *wrky6* Roots Under LP and NP Conditions

The root architecture of WT and *wrky6* seedlings differs substantially and the differences become stronger under Pi limitation conditions (Chen et al., 2009; Bakshi et al., 2015). This is reflected by different expression profiles in the roots. Here we analyze how the expression profiles of the roots of WT and *wrky6* seedlings grown on either NP or LP respond to *P. indica* colonization.

**FIGURE 1:**
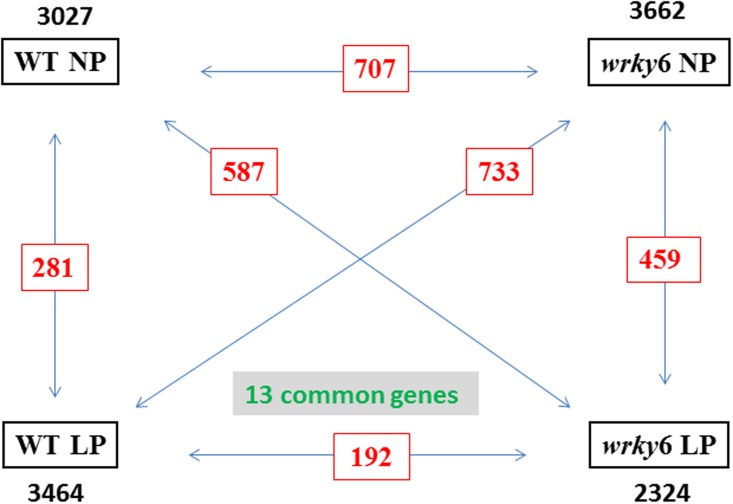
Total number of differentially regulated genes which responded to *Piriformospora indica* in the roots of WT or *wrky6* seedlings grown on either NP or LP. The black numbers show all genes regulated more than 2-fold in the roots of WT or *wrky6* seedlings grown on either NP or LP media. Common genes among the two datasets are in red. Thirteen genes are common to all datasets. For specific genes, see data submitted to NCBI.

**FIGURE 2:**
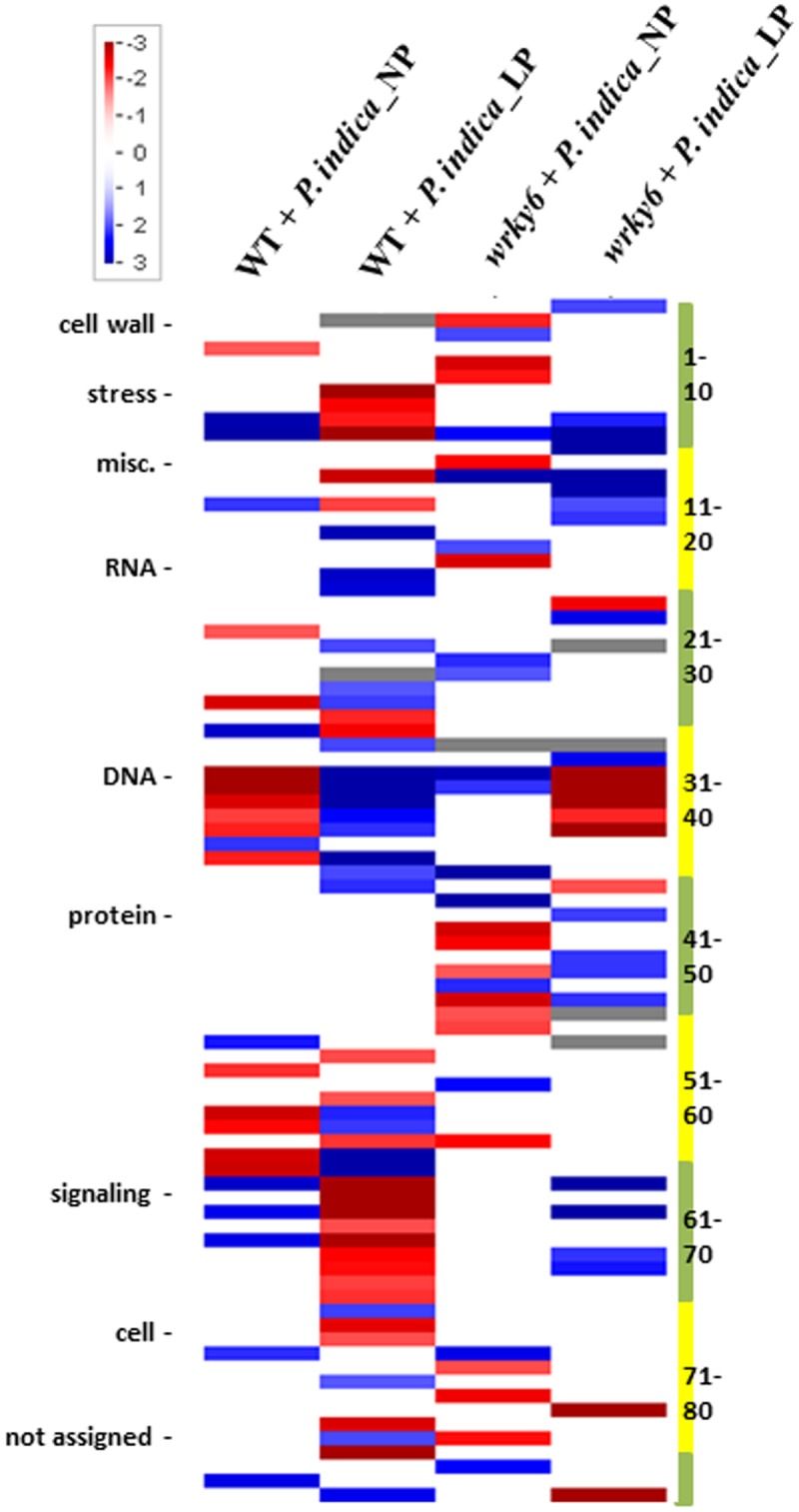
PageMan analysis of differentially expressed genes (>2-fold) by *P. indica* in WT or *wrky6* roots grown on either NP or LP. Scale depicts level of expression with blue being high and red being low. Only significantly regulated (*p*-value <0.05) categories are shown. Numbers on the right refer to the categories defined by the PageMan program, which are presented in Supplementary Table S1. For specific genes, see data submitted to NCBI. For more detailed information on the program, cf. the MapMan software at http://mapman.gabipd.org/web/guest/mapman.

**FIGURE 3:**
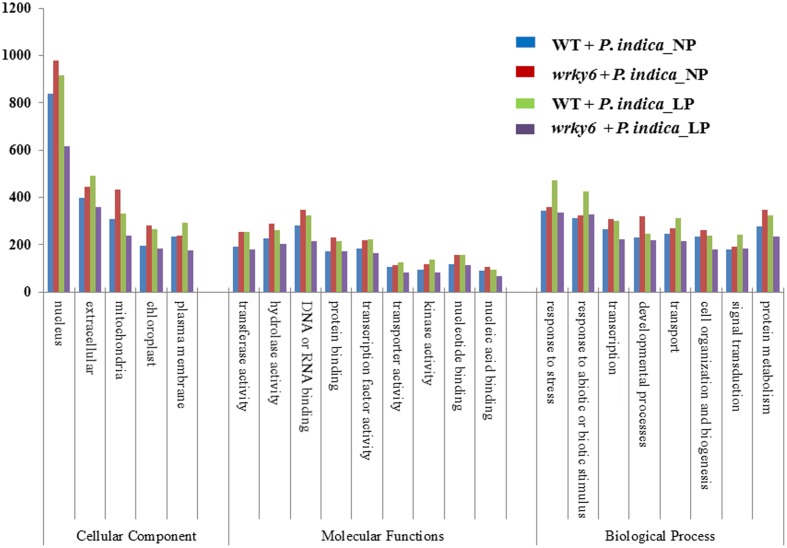
GO annotations of the genes which are differentially regulated (>2-fold) by *P. indica* in WT or *wrky6* roots grown on either NP or LP. For specific genes, see data submitted to NCBI.

**Figure 1** shows that *P. indica* affects the expression of ∼ 3000 genes (regulation > 2-fold) in WT and *wrky6* roots under NP or LP conditions. The vast majority of the responsive genes are specific for a given genotype and Pi condition, since the number of genes which are equally regulated in any comparison of the four conditions is less than 25% (**Figures 1–3**). Interestingly, only 13 genes are common in the four datasets. (for details on individual genes, cf. accession number GSE63500 at NCBI, GEO). This shows an enormous flexibility of the roots to respond to the fungus, and the response is strongly dependent on the genotype (WT vs. *wrky6* mutant) and the Pi availability (LP *vs.* NP) (**Figure 2**).

Functional categorization of the identified gene products using the *A. thaliana* Gene Ontology program (TAIR’s GO annotations; Berardini et al., 2004) demonstrates that the overall number of genes belonging to one of the four categories codes for proteins with similar functions. The strongest differences among the four categories were found for genes involved in DNA/RNA metabolism and extracellular functions, or for genes which code for mitochondrial, plastid and plasma membrane proteins. Less than 20% of these genes are common in all four categories (**Figure 3**, and GSE63500 at NCBI). Among them are hydrolases and transferases, transcription factors and numerous transporters (**Figure 3**). In each of the four datasets, different genes for proteins involved in the perception of internal and external signals, abiotic and biotic stress responses, primary protein metabolism, transcription, developmental processes and cell organization respond to *P. indica* (**Figure 3**). This is further supported by the PageMan analysis for genes differentially responding to *P. indica* in WT and *wrky6* roots under NP and LP conditions (Supplementary Figures S1–S4). For example, many DNA-related genes are down-regulated in *P. indica*-colonized WT roots under NP conditions and *P. indica*-colonized *wrky6* roots exposed to LP, but are up-regulated in colonized WT roots exposed to LP. Many genes involved in diverse signaling processes are down-regulated by *P. indica* in WT under Pi limitation, while signaling-related genes in WT roots grown under NP are up-regulated. Stress-related genes are down-regulated by *P. indica* in WT roots under Pi limitation, but up-regulated under the other three conditions (Supplementary Figures S1–S4). Categorization of the gene products according to enzyme families also demonstrates enormous differences under the four conditions, and often, the genes for one enzyme family are up-regulated under one condition and down-regulated or not regulated under other conditions (Supplementary Figure S1). Big differences can be observed for the large cytochrome P_450_ enzyme family, but also for peroxidases, phosphatases and glutathione-S-transferases. The identification of genes for GDSL lipases (Dong et al., 2016) suggests that *P. indica* also affects lipid metabolism. Finally, *P. indica* targets different members of the glucosidase gene family under NP and LP conditions and this is particularly striking for the *wrky6* mutant.

It is also obvious from Supplementary Figures S1–S4 that interfering with the Pi metabolism by either inactivating *WRKY6* or growth under Pi limitation conditions has severe consequences on many genes involved in transport processes (Supplementary Figure S2), cellular responses (Supplementary Figure S3), and regulatory functions (Supplementary Figure S4). Besides the expected effects on Pi transporters, genes for nitrate, ammonium and sulfate transporters are differentially regulated under the four conditions. We also observe big differences on genes for sugar, potassium and amino acid transporters, P- and V-ATPases and lipid transfer proteins. Genes related to calcium transport processes are up-regulated under all conditions, although to different extents (Supplementary Figure S2). Overall, the data indicate that inactivation of *WRKY6* activates biotic stress response genes under NP conditions (Supplementary Figures S3, S4). Furthermore, growth under LP conditions results in the down-regulation of many biotic-stress-related genes which are up-regulated under NP conditions, and this is observed for both WT and *wrky6* roots. The latter observation holds also true for abiotic stress-related genes (Supplementary Figure S3) and those with diverse functions (categorized as “miscellaneous”) in WT roots. Peroxiredoxin genes are preferentially down-regulated by *P. indica* in LP, and osmotic stress related genes (categorized as “drought/salt”, Supplementary Figure S3) are downregulated in the *wrky6* mutant under NP relative to the WT control. Finally, many genes related to the cell cycle are downregulated by *P. indica* in LP-grown WT seedlings when compared to those grown under NP conditions. As expected, the changes in the gene expression patterns for cellular responses (Supplementary Figure S3) are reflected by corresponding changes in regulatory pathways (Supplementary Figure S4). It is particularly striking that members of the receptor kinase gene family are downregulated by *P. indica* in LP-grown WT roots relative to roots grown under NP conditions. Taken together, the fungus activates quite different signaling pathways, as well as metabolic and developmental programs under the four conditions tested.

### *PHT1* and Pi Regulator Genes in the Arabidopsis/*P. indica* Interaction

Pi uptake from the soil and distribution of Pi within the Arabidopsis plant is mediated by nine PHT1 family members (Ayadi et al., 2015). While some *PHT1* genes are regulated in response to beneficial fungi in mycorrhizal plants, it is believed that the non-mycorrhizal plant Arabidopsis does not contain fungus-inducible *PHT1* genes (cf. Tamura et al., 2012; Sisaphaithong et al., 2012). This is consistent with our observations that most of the *PHT1* transporter genes do not respond to *P. indica* (>2-fold) under NP or LP conditions, or are only mildly regulated (*PHT1;5*; *PHT1;6* and *PHT1;8*; cf. Discussion) (**Table 1**). Furthermore, WRKY42 modulates Pi homeostasis through regulating Pi translocation and acquisition (Su et al., 2015) and the transcription factor also regulates *PHO1* expression (Su et al., 2015). WRKY45 activates *PHT1;1* expression under Pi starvation (Wang et al., 2014). Both *WRKY* genes do not respond to *P. indica* (**Table 1**). Recently, the importance of posttranscriptional processes for PHT1 proteins has been shown (Cardona-López et al., 2015), and NITROGEN LIMITATION ADAPTATION (NLA) targets Pht1;4 for degradation during the regulation of Pi homeostasis (Lin et al., 2013; Park et al., 2014). Furthermore, ESCRT-III-associated protein ALIX mediates high-affinity Pi transporter trafficking to maintain Pi homeostasis in Arabidopsis (Cardona-López et al., 2015). **Table 1** demonstrates that only *PHO1* responds to *P. indica* ( > 2-fold) in both WT and *wrky6* roots under LP, but not NP conditions. This demonstrates that *PHO1* is not only regulated under Pi limitation conditions, but also a mild target of signals from *P. indica* (cf. Discussion).

**Table 1:** Regulation of the 9 *PHOSPHATE TRANSPORTER1* (*PHT1*) genes and genes for regulatory proteins involved in *PHT1* gene and PHT1 protein regulation by *Piriformospora indica* in WT and *wrky6* roots under NP and LP conditions.

		Fold induction [colonized/uncolonized roots]			
		
Gene name	Annotation	WT NP	WT LP	*wrky6* NP	*wrky6* LP
*PHT1;1*	At5g43350	0.027 (1.02)	0.168 (1.12)	0.060 (1.04)	0.270 (1.21)
*PHT1;2*	At5g43370	-0.239 (0.85)	0.311 (1.24)	-0.365 (0.78)	0.038 (1.03)
*PHT1;3*	At5g43360	-0.046 (0.97)	0.223 (1.17)	-0.299 (0.81)	0.104 (1.08)
*PHT1;4*	At2g38940	-0.710 (0.61)	-0.471 (0.72)	-0.331 (0.80)	-0.304 (0.81)
*PHT1;5*	At2g32830	**1.656 (3.15)**	**1.184 (2.27)**	-0.221 (0.86)	0.500 (1.41)
		**[3.43 ± 0.43]**	**[2.77 ± 0.34]**		
*PHT1;6*	At5g43340	**-1.248 (0.42)**	**1.730 (3.32)**	-**1.278 (0.41)**	-0.991 (0.50)
		**[0.40 ± 0.03]**	**[3.71 ± 0.49]**	**[0.44 ± 0.04]**	**[0.42 ± 0.04]**
*PHT1;7*	At3g54700	-0.715 (0.61)	-0.125 (0.92)	0.177 (1.13)	-0.061 (0.96)
*PHT1;8*	At1g20860	1.246 **(2.37)**	-0.055 (0.96)	-0.546 (0.69)	-1.076 **(0.47)**
		**[2.13 ± 0.37]**			**[0.40 ± 0.03]**
*PHT1;9*	At1g76430	-0.194 (0.87)	-0.143 (0.91)	-0.161 (0.89)	0.064 (1.05)
*ALIX*	At1g15130	-0.066 (0.96)	0.035 (1.03)	0.188 (1.14)	0.070 (1.07)
*WRKY42*	At4g04450	0.202 (1.15)	0.349 (1.27)	-0.470 (0.72)	0.060 (1.04)
*WRKY45*	At3g01970	0.130 (1.09)	-0.716 (0.61)	0.098 (1.07)	-0.395 (0.76)
*NLA*	At1g02860	-0.134 (0.91)	0.210 (1.16)	-0.308 (0.81)	0.178 (1.13)
*PHO1*	At3g23430	0.397 (1.32)	**1.019 (2.03)**	0.250 (1.19)	**1.420 (2.68)**
			**[3.23 ± 0.26]**		**[2.90 ± 0.31]**


*The numbers represent log_*2*_ values of the mRNA levels in colonized roots vs. uncolonized roots, the numbers in brackets show fold-induction (colonized/uncolonized roots). Conditions with >2-fold regulation are in bold. The data from the microarray analysis (NCBI under the accession number GSE63500) are compared with qRT-PCR data shown in square brackets. Only those data are shown where a more than twofold regulation was observed. The primer pairs are given in Supplementary Table S1. Microarray and qRT-PCR data are based on three independent experiments, errors are SEs.*

### *P. indica* Does Not Compensate for Pi Limitation in *PHT1* Mutants

**FIGURE 4:**
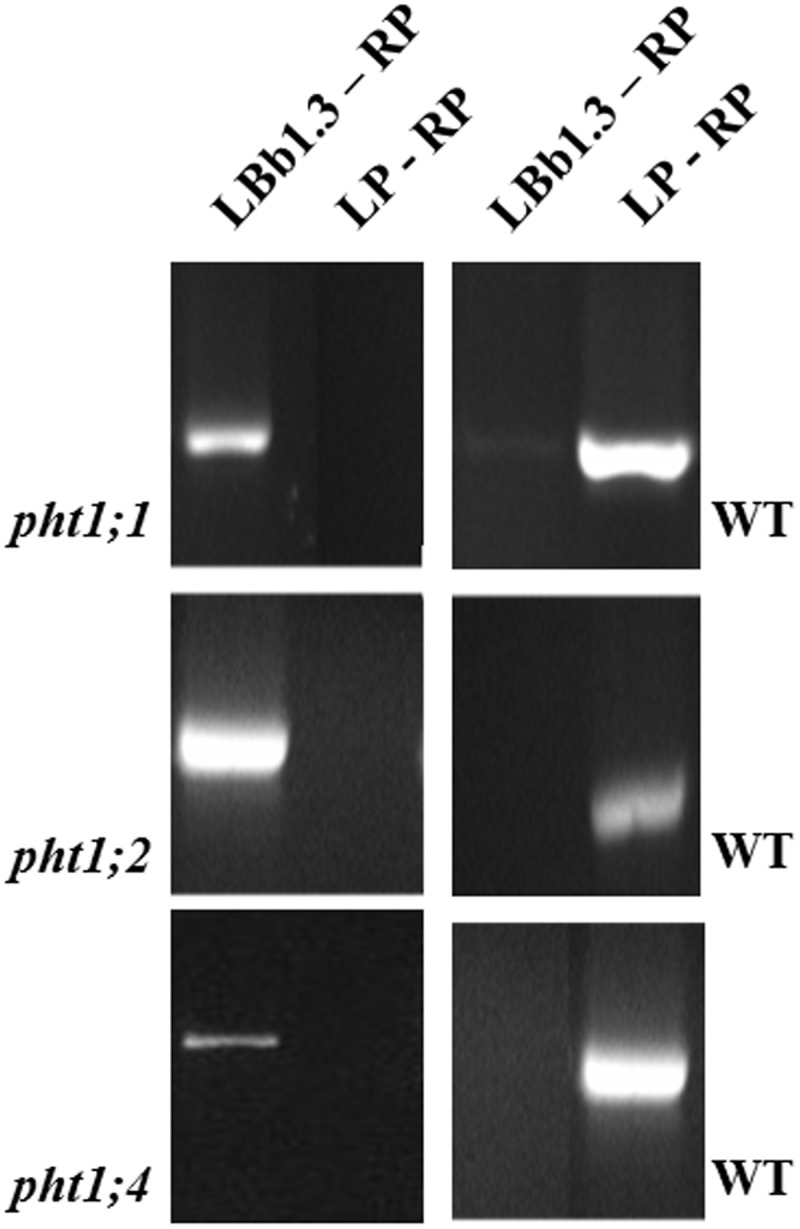
Homozygosity test for *pht1;1*, *pht1;2*, and *pht1;4* by PCR. Only one plant per knock-out line is shown. The gene-specific primers LP (left primer) and RP (right primer) amplify the expected fragments for the *PHT1;1*, *PHT1;2*, and *PHT1;4* genes in DNA preparations from WT plants, which is used as a positive control. The T-DNA specific primer LBb1.3 (TGGTTCACGTAGTGGGCCATCG) in combination with the gene-specific RPs (cf. Supplementary Table S1) amplifies a PCR fragment only when the T-DNA is inserted into the respective gene close to the RP. The PCR samples were run on a 1% agarose gel next to a size ladder and the predicted sizes of the fragments were confirmed.

Although *PHT1;1*, *PHT1;2* and *PHT1;4* do not respond (>2-fold) to *P. indica* under all tested conditions, they show the highest expression level in roots (Shin et al., 2004; Ayadi et al., 2015). Therefore, we generate homozygote knock-out lines for these three transporters, and confirmed their homozygosity using standard techniques, gene-specific primer pairs as well as a primer combination with the T-DNA insertion (Supplementary Table S2). Furthermore, *pht1;1* and *pht1;4* were crossed to generate a double knock-out line. In addition to the molecular analyses (**Figure 4**), confirmation of homozygosity for all lines can easily be tested by growth of the seedlings in the presence of arsenate (V), because this heavy metal is transported into the roots via PHT1 transporters (DiTusa et al., 2016, and references therein). After 3 weeks on MS medium with 200 μM arsenate, the fresh weight of WT seedlings was reduced by 86% compared to seedlings grown without arsenate (**Table 2**). The fresh weights of the single mutants *pht1;1* and *pht1;2* were similarly reduced (79%). This demonstrates that inactivation of these Pi transporter genes has little effect on arsenate (and probably also Pi) uptake. The performance of *pht1;4* is significantly better, suggesting that PHT1;4 is more important for arsenate (and probably Pi) uptake than PHT1;1 and PHT1;2. The fresh weight of the double knock-out line is reduced by 43% in the presence of arsenate, compared to growth without arsenate. This suggests that simultaneous inactivation of the two Pi transporters is more effective in restricting arsenate (and Pi) uptake than the effects observed for the single knock-out lines. The degree of resistance can be taken as indication for the contribution of the transporter to the Pi/arsenate uptake. Although different growth conditions and arsenate concentrations were used, our results resemble those described by Shin et al. (2004).

**Table 2:** Arabidopsis *pht1* mutants are more resistant to arsenate (V) than the WT. After 10 days, the seedlings were transferred to MS medium with 1 mM Pi and 200 μM arsenate.

Plants	Fresh weight (mg) (+ arsenate)	Fresh weight (mg) (– arsenate)	% inhibition of fresh weight (%)
WT	19.4 ± 1.1	138.6 ± 7.1	86
*pht1;1*	25.5 ± 3.1	121.4 ± 6.5	79
*pht1;2*	24.4 ± 2.3	116.1 ± 5.4	79
*pht1;4*	32.5 ± 3.9	105.8 ± 5.2	69
*pht1;1 pht1;4*	51.1 ± 2.9	89.6 ± 2.4	43


*The fresh weights of 3-week-old Arabidopsis WT, *pht1;1*, *pht1;2*, *pht1;4* and *pht1;1 pht1;4* single and double mutants are given. % fresh weight is calculated relative to the fresh weight of the seedlings grown without arsenate. Based on three independent experiments with 30 seedlings each, errors are SEs.*

**FIGURE 5:**
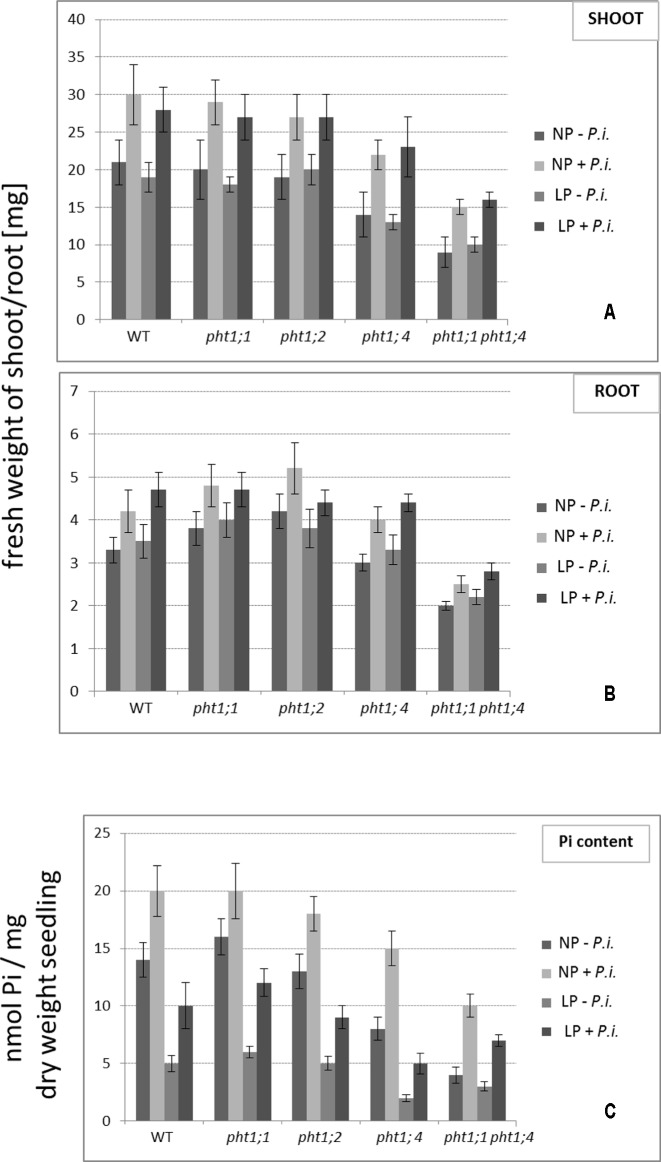
The effect of *P. indica* on the fresh weights **(A,B)** and Pi content **(C)** of WT and *pht1* mutant seedlings grown on NP and LP medium. Seedlings were transferred to PNM medium with either NP or LP and with or without *P. indica* and the shoots and roots were harvested separately after 2 weeks. Based on five independent co-cultivation experiments with 60 seedlings per treatment and genotype. Bars represent SEs.

WT seedlings and all mutants and were grown on NP and LP medium in the absence or presence of *P. indica* for 2 weeks. Shoots and roots were harvested and weighed (**Figures 5A,B**). The fresh weights of the shoots and roots of the *pht1;1* and *pht1;2* seedlings did not differ significantly from those of the WT, the weights of the shoots and roots of the *pht1;4* seedlings were slightly reduced and those of the double knock-out line ∼ half of the weights of the WT. Only little differences can be observed for seedlings grown on NP or LP medium. This might be due to the pre-cultivation of the seedlings on full MS medium: the Pi that is taken up during this period might be sufficient for the seedling’s growth during the next 2 weeks. In all cases, we observed an increase in the shoot and root fresh weights for seedlings grown in the presence of *P. indica*. However, the relative increases (% increase) among the different mutant and WT seedlings are comparable. Thus, *P. indica* does not compensate for the absence of specific PHT1s by transferring more Pi from the soil to the roots. This is particularly striking for the double knock-out mutant. Although the weight of the mutant is approximately half of the weight of a WT seedling, the % increases in their fresh weights induced by *P. indica* are approximately the same (∼ 20%). This indicates that *P. indica* promotes growth of all seedlings grown under LP and NP conditions, and this is independent of the presence or absence of the tested PHT1 transporters.

To test whether *P. indica* participates in Pi allocation, we used the same growth conditions and measured the total Pi content in the seedlings (**Figure 5C**). Similar to previous reports (Shahollari et al., 2005), we observed that all *P. indica*-exposed seedlings contained more Pi than the seedlings not exposed to the fungus. Closer inspection of the data shows that the % increase in the Pi content is not different between WT and mutant seedlings. This again is consistent with the idea that *P. indica* does not compensate for the lower Pi uptake of the Pi mutants. The higher Pi content in colonized seedlings might be caused by better access to the nutrient in the presence of the hyphal mycelium, and/or because Pi from the fungal hyphae is delivered to the host.

Since the double knock-out line showed the strongest phenotype, we grew the seedlings under LP conditions in the absence or presence of *P. indica* and tested whether the mutant respond to Pi limitation by activating Pi starvation-response and transporter genes and whether *P. indica* has an effect on the regulation of these genes in the mutant. **Table 3** shows that the expression of several unrelated Pi starvation-response and transporter genes are up-regulated in the double mutant under LP and that their expression is not significantly affected by *P. indica*. This includes *PHOSPHATE STARVATION RESPONSE1* (*PSR1*) which codes for a MYB transcription factor involved in the Pi starvation response (Rubio et al., 2001), *PHOSPHATE STARVATION RESPONSE1* (*PHR1*), a master regulator of Pi homeostasis (e.g., Linn et al., 2017), which balances between nutrition and immunity in plants (Motte and Beeckman, 2017), the gene for the zinc-finger transcription factor ZAT6, which controls Pi homeostasis in roots (Devaiah et al., 2007a), for MYB62 involved in Pi homeostasis and hormone actions (Devaiah et al., 2009) and for WRKY45 which activates *PHT1;1* expression in response to Pi starvation (Wang et al., 2014). Thus, the fungus does not interfere with the regulation of these genes in the LP-grown *pht1;1 pht1;4* mutant.

**Table 3:** Pi starvation response-genes are up-regulated in LP-grown *pht1;1 pht1;4* mutant seedlings, in the presence and absence of *P. indica*.

Gene	WT, LP,- *P. indica*	WT, LP,+ *P. indica*	*pht1;1 pht1;4*, LP, – *P. indica*	*pht1;1 pht1;4*, LP, + *P. indica*
*WRKY45*	1.00 ± 0.2	0.80 ± 0.09	2,20 ± 0,25	2.32 ± 0,19
*PHT1;5*	1.00 ± 0,12	0.77 ± 0.11	2.33 ± 0,27	2,40 ± 0,23
*PHR1*	1.00 ± 0,21	0.93 ± 0.09	1.96 ± 0,24	2,11 ± 0,26
*ZAT6*	1.00 ± 0,27	1.11 ± 0.12	2.45 ± 0,20	2,20 ± 0,17
*MYB62*	1.00 ± 0,11	1.06 ± 0.07	2.66 ± 0,21	2.45 ± 0,22


*WT and double knock-out seedlings were grown in LP for 2 weeks in the presence or absence of *P. indica* before RNA extraction for qRT-PCR. Primer pairs for the analyzed genes are given in Supplementary Table S1. The mRNA level of a given gene from WT seedlings grown under LP without *P. indica* was set as 1.0 and the other values are expressed relative to it. For additional information about the genes not discussed in this paper: *PHR1* (Rubio et al., 2001), ZAT6 (Devaiah et al., 2007a), and *MYB62* (Devaiah et al., 2009). Based on three independent RNA extraction and two technical repetitions per RNA. Errors are SEs.*

**FIGURE 6:**
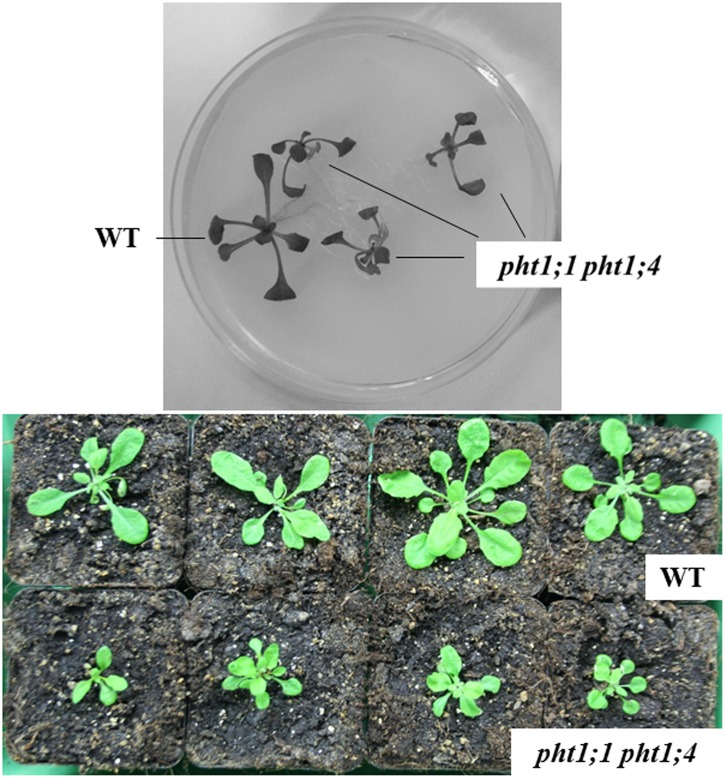
Phenotype of WT and *pht1;1 pht1;4* seedlings/plants. Top: After 10 days, WT and *pht1;1 pht1;4* seedlings were transferred to PNM medium with NP for 3 weeks. Bottom: Arabidopsis WT (upper 4 plants) and *pht1;1 pht1;4* double knock-out lines (lower 4 plants) were grown in Petri dishes for 10 days before transfer to soil for additional 4 weeks. Growth occurred in a greenhouse.

The double mutant is impaired in long-term growth experiments on natural soil (**Figure 6**). After germination and initial growth in Petri dishes for 10 days, the seedlings were transferred to soil for additional 3 weeks. We observed an approximately 50% reduction in the fresh weight compared to WT control plants (**Figure 6** lower panel). This suggests the combination of the two transporters is important for growth on natural soil.

**FIGURE 7:**
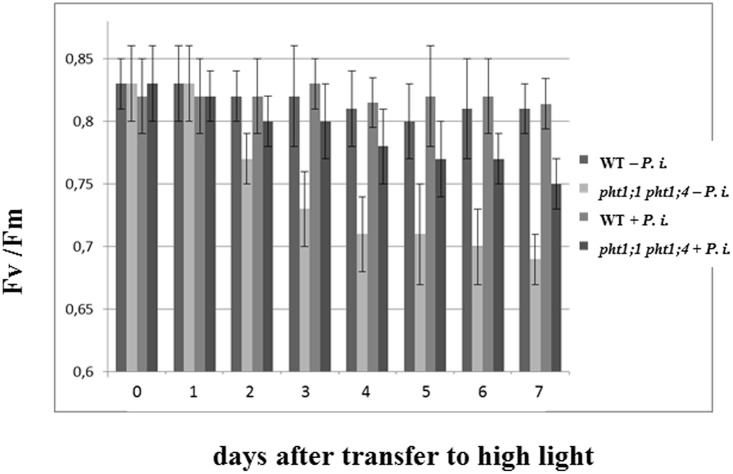
*Piriformospora indica* promotes the efficiency of the photosynthetic electron transport of the double knock-out line exposed to high light stress under Pi limitation. Chlorophyll fluorescence measurements (Fv/Fm) for *P. indica*-exposed WT and *pht1;1 pht1;4* seedlings which were transferred to fresh PNM plates with LP and high light intensity (300 μmol m^-2^ s^-1^) for 1 week (cf. Materials and Methods). Based on three independent experiments with 16 plants each. Bars represent SEs.

To test whether *P. indica* promotes the performance of the double knock-out line, we transferred the seedlings to PNM medium and high light intensity (300 μmol m^-2^ s^-1^). The chlorophyll fluorescence parameters Fv/Fm showing the efficiency of the photosynthetic electron transport were measured daily under LP and NP conditions over a period of 7 days (**Figure 7**). Under NP conditions, no difference between colonized and uncolonized WT and double knock-out seedlings can be detected (data not shown). However, under LP conditions, a decline in the Fv/Fm values can be detected for uncolonized *pht1;1 pht1;4* seedlings, and this stress response is partially compensated in the presence of the fungus. Thus, *P. indica* partially promotes the performance of the double knock-out line under Pi limitation conditions.

## Discussion

### The Genotype and Pi Availability Have a Strong Influence on *P. indica*-Targeted Genes

Previous interaction studies of root-colonizing microbes with plants have shown that the mutualistic interaction and the benefits for the two partners is strongly dependent on environmental conditions (e.g., Pánková et al., 2011; Moeller et al., 2014) and differ between plant species when they are colonized by the same microbe (Lee et al., 2011; Lahrmann et al., 2013). Here, we compare a WT Arabidopsis line with a mutant impaired in the WRKY6 transcription factor which is a central regulator of Pi metabolism in Arabidopsis (Hamburger et al., 2002; Liu et al., 2012). By growing these seedlings under NP and LP conditions, we show that *P. indica* targets quite different genes in both genotypes and under the two Pi conditions. Genotype-dependent alterations in gene expression profiles in response to various biotic and abiotic stresses have been described for many ecotypes, varieties, lines and mutants impaired in regulatory loci. Genotype-specific expression of miRNAs might explain distinct cold (Zhang et al., 2014) or salt sensitivities (Ding et al., 2009; Beritognolo et al., 2011; Yin et al., 2012). Genotype-specific defense gene expression programs were reported for two cultivars of *Glycine max* (Klink et al., 2011). In *Sorghum bicolor* a genotype-specific expression atlas for vegetative tissues was published (Shakoor et al., 2014). Ashraf et al. (2009) described comparative analyses of genotype-dependent expressed sequence tags and stress-responsive transcriptome for chickpea wilt. Here, we show that a mutation in the *WRKY6* gene which strongly affects plant performance under Pi limitation conditions, results in a severe reprogramming of the root transcriptome after *P. indica* infection. The altered gene expression profile affects many biochemical, signaling and metabolic processes, which are not related to the Pi metabolism (**Figures 2**, **3** and Supplementary Figures S1–S4). To our knowledge this is the first report on a comprehensive analysis of gene reprogramming in response to *P. indica* in roots of two genotypes, the WT and the *wrky6* mutant. The huge difference in the expression profiles clearly indicates that the fungus targets different genes and consequently induces completely different physiological responses in the roots of WT and *wrky6* seedlings under the two different Pi conditions. This might have important implication for the application of the fungus in agriculture, since its interaction with different cultivars of a crop plant might differ and this again is dependent on environmental and soil conditions.

Analysis of genes involved in Pi starvation have been analyzed in many plant species (e.g., Nilsson et al., 2010). Interesting in the study performed here the number of *P. indica* stimulated genes in roots of both WT and *wrky6* seedlings is quite different in LP compared to NP conditions Not only Pi-related signaling and metabolic pathways, but also developmental programs, defense strategy and transport processes are affected in WT and *wrky6* roots. Under our standardized conditions, ∼70–80% of the plant genes are differentially expressed in response to *P. indica* by inactivation of *WRKY6* and/or change in the amount of available Pi (**Figure 2**). We assume that similar phenomena could be observed for agriculturally important crops interacting with beneficial root-colonizing fungi. Therefore, the interplay between root-colonizing microbes with different cultivars, the soil quality and fertilizations may have a strong influence on the root metabolism.

### *PHT1* Genes and *P. indica*

The members of the AtPHT1 protein family share a high degree of similarity with overlapping expression patterns (Ayadi et al., 2015). PHT1;1 and PHT1;4 form homomeric and heteromeric complexes (Fontenot et al., 2015) and both transporters play a major role in Pi acquisition from low- and high-Pi environments (Shin et al., 2004). Their genes show the highest expression of all *PHT1* genes in Arabidopsis roots (Shin et al., 2004), but PHT1;1 also plays an important role in Pi translocation from roots to leaves in high Pi conditions (Ayadi et al., 2015). PHT1;2 cooperates with PHT1;1 and PHT1;4 in Pi uptake from the soil (Ayadi et al., 2015) and *PHT1;6* is mainly expressed in flower tissue (Karandashov et al., 2004). Members of the *PHT1* gene family are up-regulated by mycorrhizal fungi in mycorrhiza-forming plant species (e.g., Ceasar et al., 2014; Walder et al., 2015; Kariman et al., 2016; for some recent publications), however the Arabidopsis homologs (*pht1;5*, *pht1;6*, and *pht1;8*) do not or barely respond to *P. indica.* The slight response of *pht1;5* to *P. indica* might be related to the role of PHT1;5 as mobilizer of Pi between source and sink organs (Nagarajan et al., 2011). Under low Pi conditions, *pht1;5* mutants show reduced Pi allocation to the shoots and elevated transcript levels for several Pi starvation-response genes (Nagarajan et al., 2011). *P. indica* might interfere with the translocation and regulation of Pi under Pi limitation. Karandashov et al. (2004) identified six *cis*-regulatory elements which are present in different combinations and numbers in mycorrhiza-inducible *PHT1* genes from different plant species. None of these elements were found in the *P. indica*-responsive *AtPHT1;5* and *AtPHT1;6* promoters, but four of them are present in the root-specific *AtPHT1;8* promoter which shows a low response to *P. indica* (**Table 1**). Whether these elements are involved in *P. indica* -mediated *PHT1;8* expression, is unclear.

Co-cultivation of *pht1* mutants with *P. indica* demonstrates that the fungus does not compensate for Pi shortage by stimulating the expression of specific *PHT1* family members (**Table 1** and **Figure 5**) or promoting Pi uptake via other ways. The % growth promotion and Pi uptake in the presence of the fungus is comparable for WT and mutant seedlings. Even for the double knock-out line, the positive effects of the fungus are comparable to the WT (**Figure 5**). Together with the observation that the *PHT1* genes are not or barely regulated by *P. indica*, it is conceivable that the fungus targets additional genetic programs, which are not directly related to Pi availability.

### PHO1 Respond to *P. indica*

Besides *PHT1* genes (Wasaki et al., 2003; Wu et al., 2003; Misson et al., 2005), the mRNA levels for several transcription factors involved in controlling Pi homeostasis, such as AtPHR1 (Rubio et al., 2001), the rice Pi starvation-induced transcription factor1 (Yi et al., 2005), AtWRKY75 (Devaiah et al., 2007a), the Arabidopsis zinc finger family member 6 (AtZAT6; Devaiah et al., 2007b), AtMYB62 (Devaiah et al., 2009), and AtWRKY6 (Chen et al., 2009) are upregulated under Pi limitation in different plant species (Wang et al., 2014). Also, PHO1 has been shown to be an important regulator in controlling Pi homeostasis (Chen et al., 2007). We observed a mild, but significant upregulation of the *PHO1* mRNA level by *P. indica* in LP-exposed Arabidopsis roots (**Table 1**). PHO1 plays an important role in Pi translocation from roots to shoots (Poirier et al., 1991; Wang et al., 2014), is located primarily in root stellar cells and controls Pi xylem loading from root stellar cells (Hamburger et al., 2002). Thus, the protein is mainly involved in long distance Pi transport in Arabidopsis (Su et al., 2015). The *pho1* mutant is impaired in loading Pi to the xylem vessel in the roots (Poirier et al., 1991). Taken together, our data suggest that *P. indica* interferes primarily with the Pi distribution and metabolism in Arabidopsis under Pi limitations rather than by promoting Pi uptake from the soil.

## Author Contributions

MB: performed most of the experiments, except those described for others authors. IS: generated the ko lines. DM: analysed microarray data, performed low Pi experiments with Arabidopsis. JT: performed Pi experiments with the double mutant. AJ: supervision of Ph.D. students together with RO (common India-Germany DAAD project). AV: supervision of Ph.D. students together with RO (common India-Germany DAAD project). K-WY: supervision of microarray analyses. RO: supervision of Ph.D. students together with RO (common India-Germany DAAD project)

## Conflict of Interest Statement

The authors declare that the research was conducted in the absence of any commercial or financial relationships that could be construed as a potential conflict of interest.

## Footnotes

1 http://pubman.mpdl.mpg.de/pubman/item/escidoc:1587455/component/escidoc:1674163/IMPRS056.pdf

2 www.arabidopsis.org

3 http://mapman.gabipd.org/web/guest/mapman

4 http://www.clcbio.com/products/clc-main-workbench

## Abbreviations

LP: low phosphate concentration
NP: normal phosphate concentration
Pi: phosphate
WT: wild-type
