# Supplementary material for: Piriformospora indica Reprograms Gene Expression in Arabidopsis Phosphate Metabolism Mutants But Does Not Compensate for Phosphate Limitation
Source: Front Microbiol. 2017 Jul 12;8:1262. doi: 10.3389/fmicb.2017.01262 (PMC5506084; doi:10.3389/fmicb.2017.01262)
Supplement: Supplementary file 1 [file Table_1.PDF]

**Supplementary Table 1**

Categories of genes differentially regulated in response to *P. indica* colonization in WT and *wrky6* roots, as well as on NP or LP media. Numbers refer to the Mapman analysis shown in Figure 3. (Numbers in blocks of 10 are given on the right site of the figure.)

| **Number** | **Category according to Mapman** |
|---|---|
| **1** | **minor CHO metabolism, raffinose family** |
| **2** | **glycolysis** |
| **3** | **cell wall** |
| **4** | **amino acid metabolism, synthesis** |
| **5** | **hormone metabolism, ethylene** |
| **6** | **hormone metabolism, ethylene, synthesis, degradation** |
| **7** | **stress** |
| **8** | **biotic stress** |
| **9** | **abiotic stress** |
| **10** | **abiotic stress, unspecific** |
| **11** | **misc.** |
| **12** | **misc., gluco-, galacto-, manosidases** |
| **13** | **misc., nitrilase, nitril hydrolase, barberine bridge enzymes** |
| **14** | **misc., gluthatione-S-transferases** |
| **15** | **misc., cytochrome P450 enzymes** |
| **16** | **misc., peroxidases** |
| **17** | **misc., acid and other phosphatases** |
| **18** | **misc., plastocyanin-like proteins** |
| **19** | **misc., protease inhibitors, seed storage/lipid transfer proteins** |
| **20** | **RNA** |
| **21** | **RNA regulation of transcription** |
| **22** | **RNA regulation of transcription, AP2, EREBP, Apelata2, ethylene transcription factor** |
| **23** | **RNA, regulation of transcription, ARF, auxin responsive factors** |
| **24** | **RNA, regulation of transcription, bHLH** |
| **25** | **RNA, regulation of transcription, C2C2(Zn), YABBY family members** |
| **26** | **RNA, regulation of transcription, HB, homeobox transcription factors** |
| **27** | **RNA, regulation of transcription, HSF, heat shock transcription factors** |
| **28** | **RNA, regulation of transcription,MADS box transcription factors** |
| **29** | **RNA, regulation of transcription, MYB domain transcription factors** |
| **30** | **RNA, regulation of transcription, NAC domain transcription factors** |
| **31** | **RNA, regulation of transcription, WRKY domain transcription factors** |
| **32** | **RNA, regulation of transcription, methyl-binding domain proteins** |
| **33** | **RNA, regulation of transcription, putative transcription factors** |
| **34** | **DNA** |
| **35** | **DNA, synthesis, chromatin structure** |
| **36** | **DNA, synthesis, chromatin structure, retrotransposons/transposase** |
| **37** | **DNA, synthesis, chromatin structure, retrotransposons/transposase** |
| **37** | **DNA, synthesis, chromatin structure, retrotransposons/transposase** |
| **39** | **DNA, synthesis, chromatin structure, retrotransposons/transposase** |
| **40** | **DNA, synthesis, chromatin structure, retrotransposons/transposase** |
| **41** | **DNA, synthesis, chromatin structure, retrotransposons/transposase** |
| **42** | **DNA, synthesis, chromatin structure, retrotransposons/transposase** |

| | |
|---|---|
| **43** | **DNA unspecific** |
| **44** | **protein** |
| **45** | **protein synthesis, ribosome biosynthesis** |
| **46** | **protein synthesis, pre-tRNA processing enzymes** |
| **47** | **protein synthesis, tRNA** |
| **48** | **protein synthesis, tRNA nucleus** |
| **49** | **protein synthesis, tRNA nucleus, tRNA-Arg** |
| **50** | **protein synthesis, tRNA nucleus, tRNA-Cys** |
| **51** | **protein synthesis, tRNA nucleus, tRNA-His** |
| **52** | **protein synthesis, tRNA nucleus, tRNA-Ile** |
| **53** | **protein targeting, nucleus** |
| **54** | **protein, posttranslational modifications** |
| **55** | **protein, degradation** |
| **56** | **protein, degradation, aspartate protease** |
| **57** | **protein, degradation, AAA-type** |
| **58** | **protein, degradation, ubiquitin** |
| **59** | **protein, degradation, ubiquitin, E3** |
| **60** | **protein, degradation,  ubiquitin, E3, RING** |
| **61** | **protein, degradation, ubiquitin, E3, SCF** |
| **62** | **protein, degradation, ubiquitin, E3, SCF, F-Box** |
| **63** | **signaling** |
| **64** | **signaling in sugar and nutrient metabolism** |
| **65** | **signaling, receptor kinases** |
| **66** | **signaling, receptor kinases, leucine-rich repeat proteins** |
| **67** | **signaling, receptor kinases, DUF26** |
| **68** | **signaling, receptor kinases, legume, lectin** |
| **69** | **signaling, receptor kinases, S-locus glycoprotein-like** |
| **70** | **signaling, receptor kinases,wall associated kinases** |
| **71** | **signaling, receptor kinases, misc.** |
| **72** | **signaling, misc.** |
| **73** | **cell** |
| **74** | **cell organisation** |
| **75** | **development, storage proteins** |
| **76** | **transport, metabolite transporters at the envelope membrane** |
| **77** | **transport, potassium** |
| **78** | **transport, unspecific anions** |
| **79** | **not assigned** |
| **80** | **not assigned, no ontology** |
| **81** | **not assigned, no ontology, pentatricopeptide repeat-containing proteins** |
| **82** | **not assigned, no ontology, DC1 domain-containing proteins** |
| **83** | **not assigned, no ontology, glycine-rich protein** |
| **84** | **not assigned, no ontology, proline-rich protein** |
| **85** | **not assigned, unknown** |
