# Supplementary material for: Piriformospora indica Reprograms Gene Expression in Arabidopsis Phosphate Metabolism Mutants But Does Not Compensate for Phosphate Limitation
Source: Front Microbiol. 2017 Jul 12;8:1262. doi: 10.3389/fmicb.2017.01262 (PMC5506084; doi:10.3389/fmicb.2017.01262)
Supplement: Supplementary file 2 [file Table_2.DOCX]

**Suppl. Tab. 2**

The following primer pairs were used for the real-time PCR analyses:

PHT1;1_forw GGGTCTTCTTGTTTGCGTA

PHT1;1_rev TCTTGTGCTCTGTCGCTT

PHT1;2_forw GCTTCTGGCCTCTCTTTT

PHT1;2_rev GGGTCTTCTTGTTTGCGT

PHT1;3_forw ACAAACTCGGACGGAAAAA

PHT1;3_rev AACTGCAAGTGCCACAAAA

PHT1;4_forw TTTTCGGTTTTGGCTTGG

PHT1;4_rev ATCAGCTTGAGGAATCGT

PHT1;5_forw GAAGGTGAAATTAGGGTTTG

PHT1;5_rev GGATGAAAATGCCGGAAA

PHT1;6_forw GAGGAGGAACCATGTGGA

PHT1;6_rev GGATGAAGATGCCCGAAA

PHT1;7_forw AAGGCGATTAGGGTTTGT

PHT1;7_rev AGGTTTTGCAAGTGGAGA

PHT1;8_forw GGAGGTTGCTTGTGTAGA

PHT1;8_rev GGCCTGGAGACTTATACTT

PHT1;9_forw GGAAACGGCGAGAGAAGA

PHT1;9_rev GGATATAGCGTGGAGGTTGA

ALIX_forw TCCTTGATCTCTCGATAC

ALIX_rev GGCTCCTATGAAGATATG

WRKY42_forw ATGGTGGATGATGGATTG

WRKY42_rev GGAATTGAAGTTGTTGGTTG

WRKY45_forw GTGGAAATTATCAGAGGG

WRKY45_rev GAAGAAGAAGAAAGAGAGAG

NLA_forw TTCTCCTCCTCCTCCTGT

NLA_rev GTTGCTTCTGCGGTTTTC

PHO1_forw TCTTCCGTTTTTGCAGGT

PHO1_rev CGTCTTGATATGCCCATT

ACTIN2_ forw ggaatccacgagacaaccta

AcTIN2_ re ATCTTCATGCTGCTTGGTGC

PHR1_ forw GGTAAGACCAGAGTTTTG

PHR1_re GGCCAGAACCATCAGAAA

ZAT6_forw GCCTCCGTTCTTTCCTTC

ZAT6_re GCCTCCGTTCTTTCCTTC

MYB62_forw GTTGTAGATTGAGATGGTTG

MYB62-re TCGTTATCCGTTCTTCCT
