# Supplementary material for: Piriformospora indica Reprograms Gene Expression in Arabidopsis Phosphate Metabolism Mutants But Does Not Compensate for Phosphate Limitation
Source: Front Microbiol. 2017 Jul 12;8:1262. doi: 10.3389/fmicb.2017.01262 (PMC5506084; doi:10.3389/fmicb.2017.01262)

Supplementary Figure 1
(ENZYME FAMILIES)

1-1 *wrky*6 + *P. indica*_LP
1-2 *wrky*6 + *P. indica*_NP
1-3 WT + *P. indica*_LP
1-4 WT + *P. indica*_NP

Mapman pathway depicting differentially expressed genes (2-fold) by *P. indica* in WT or *wrky6* roots grown on either NP or LP. (**Suppl. Fig. 1**) Genes coding for enzyme families, (**Suppl. Fig. 2**) for transporter families, (**Suppl. Fig. 3**) for different cellular responses and (**Suppl. Fig. 4**) for regulatory processes. Level of expression with blue is high and red is low. For more detailed information, cf. the MapMan software at http://mapman.gabipd.org/web/guest/ mapman.

Enyzme families: (1) *wrky*6 + *P. indica*_LP

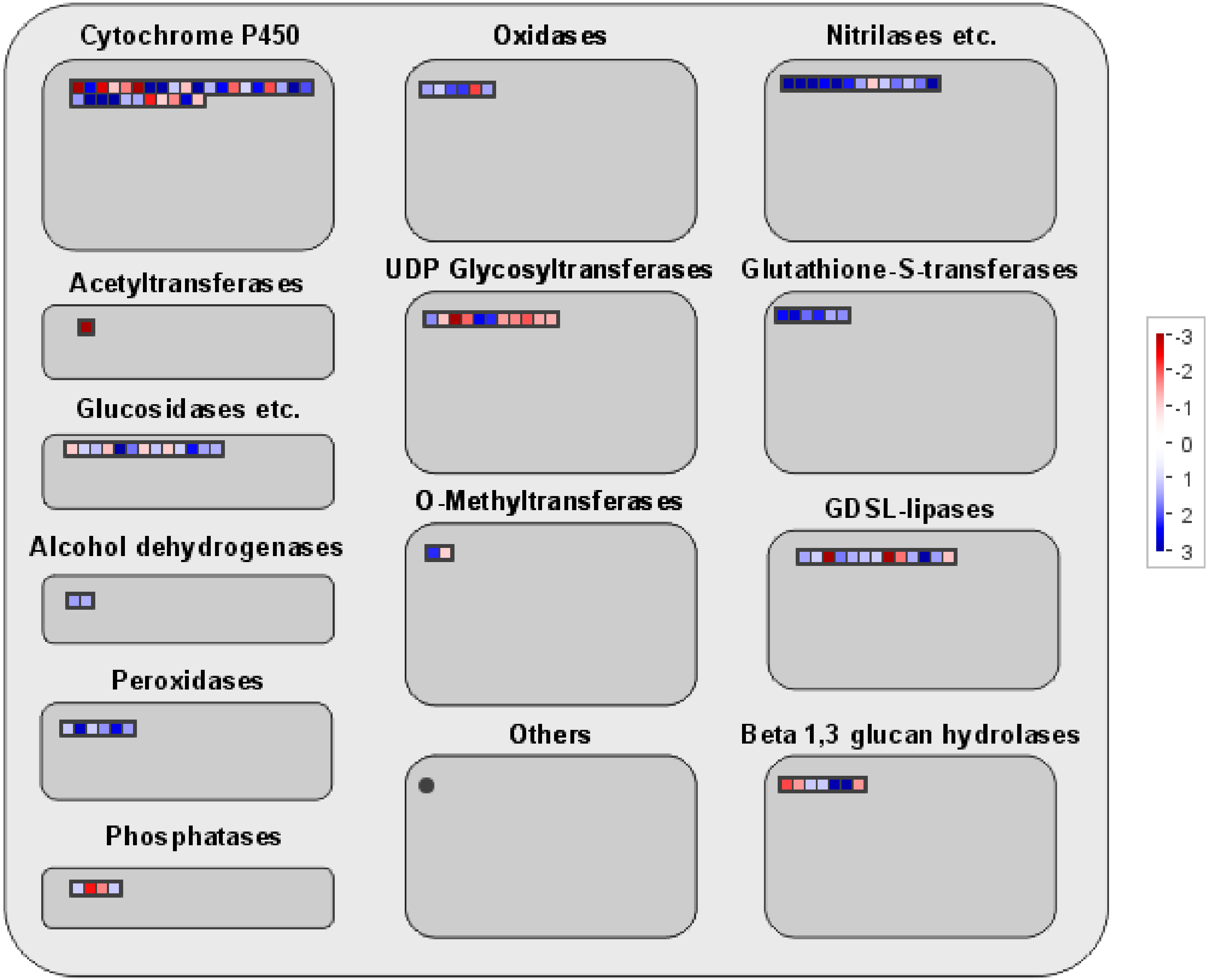

Enyzme families: (2) *wrky*6 + *P. indica*_NP

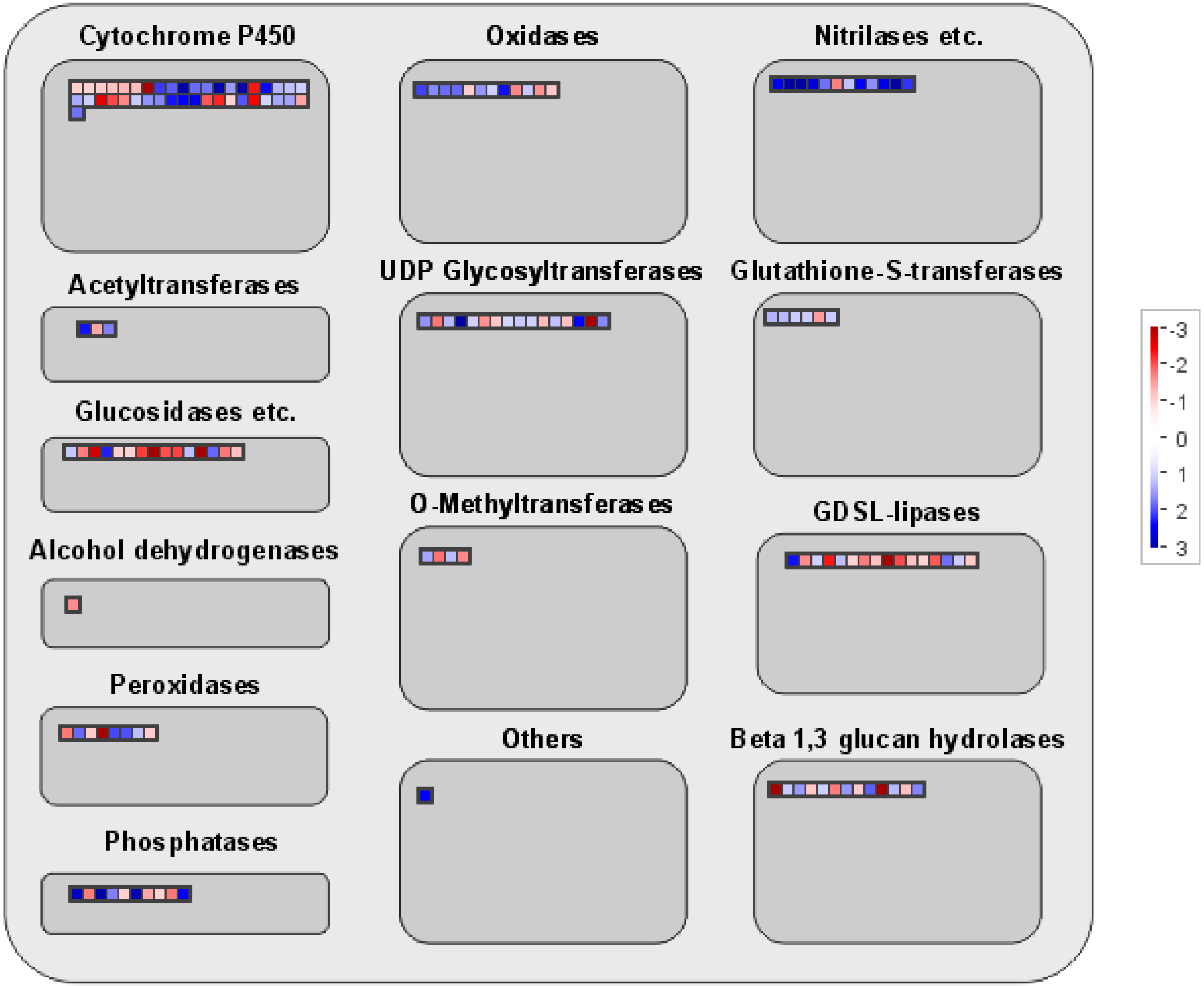

Enyzme families: (3) WT+ *P. indica*_LP

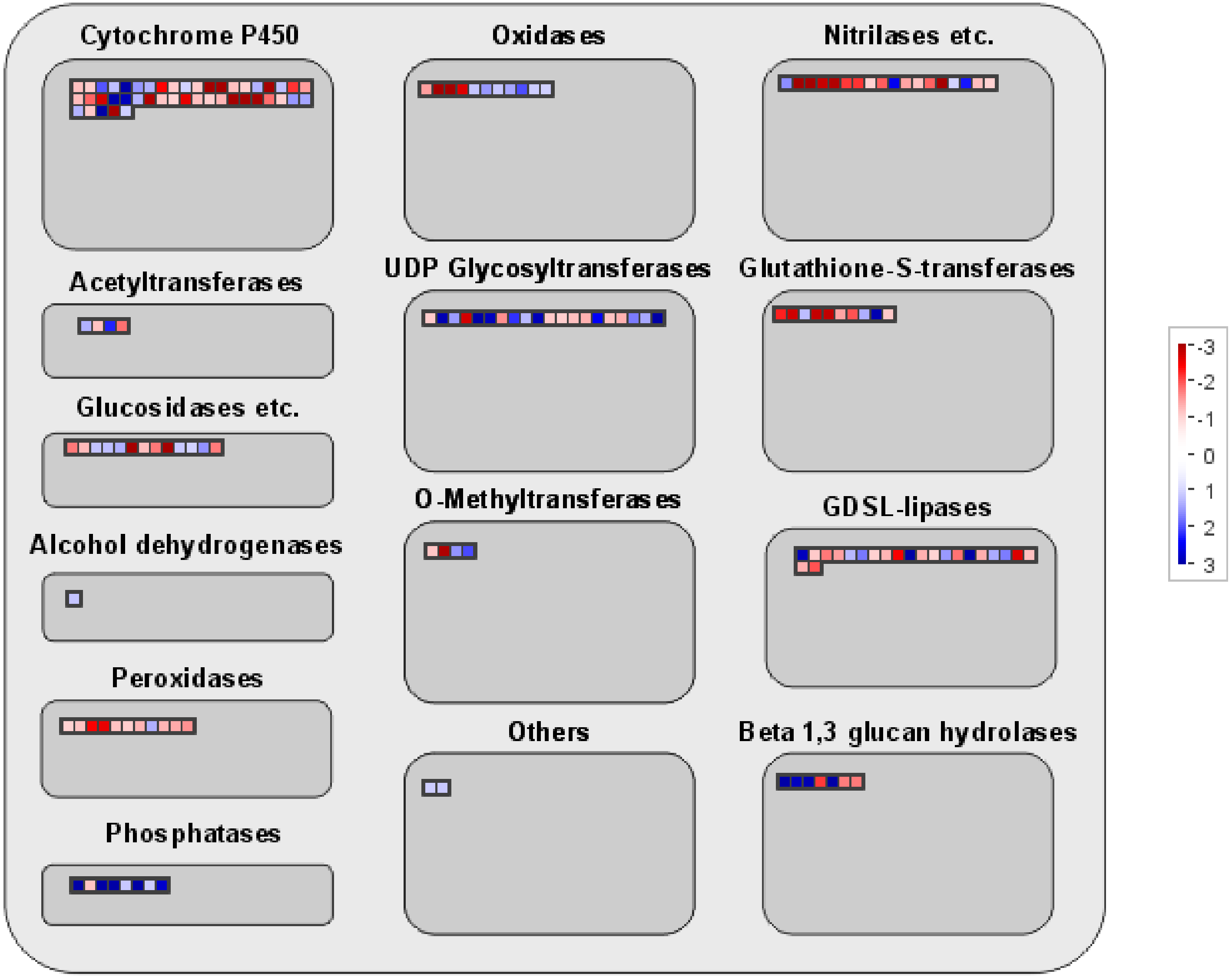

Enyzme families: (4) WT+ *P. indica*_NP

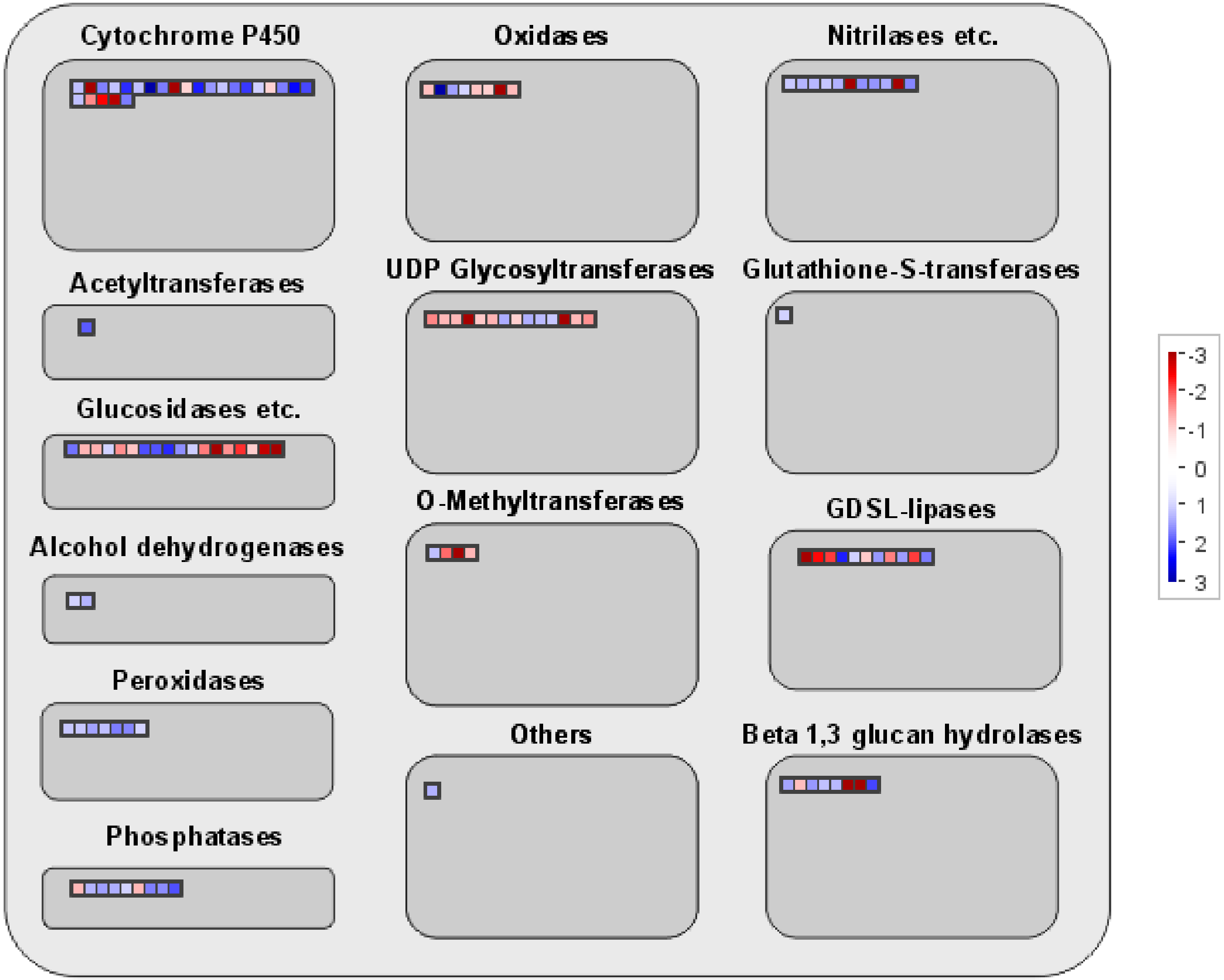

Supplementary Figure 2 (TRANSPORTER)

2-1 *wrky*6 + *P. indica*_LP
2-2 *wrky*6 + *P. indica*_NP
2-3 WT + *P. indica*_LP
2-4 WT + *P. indica*_NP

Mapman pathway depicting differentially expressed genes (2-fold) by *P. indica* in WT or *wrky6* roots grown on either NP or LP. (**Suppl. Fig. 1**) Genes coding for enzyme families, (**Suppl. Fig. 2**) for transporter families, (**Suppl. Fig. 3**) for different cellular responses and (**Suppl. Fig. 4**) for regulatory processes. Level of expression with blue is high and red is low. For more detailed information, cf. the MapMan software at http://mapman.gabipd.org/web/guest/ mapman.

Transporter: (1) *wrky6* + *P. indica*_LP

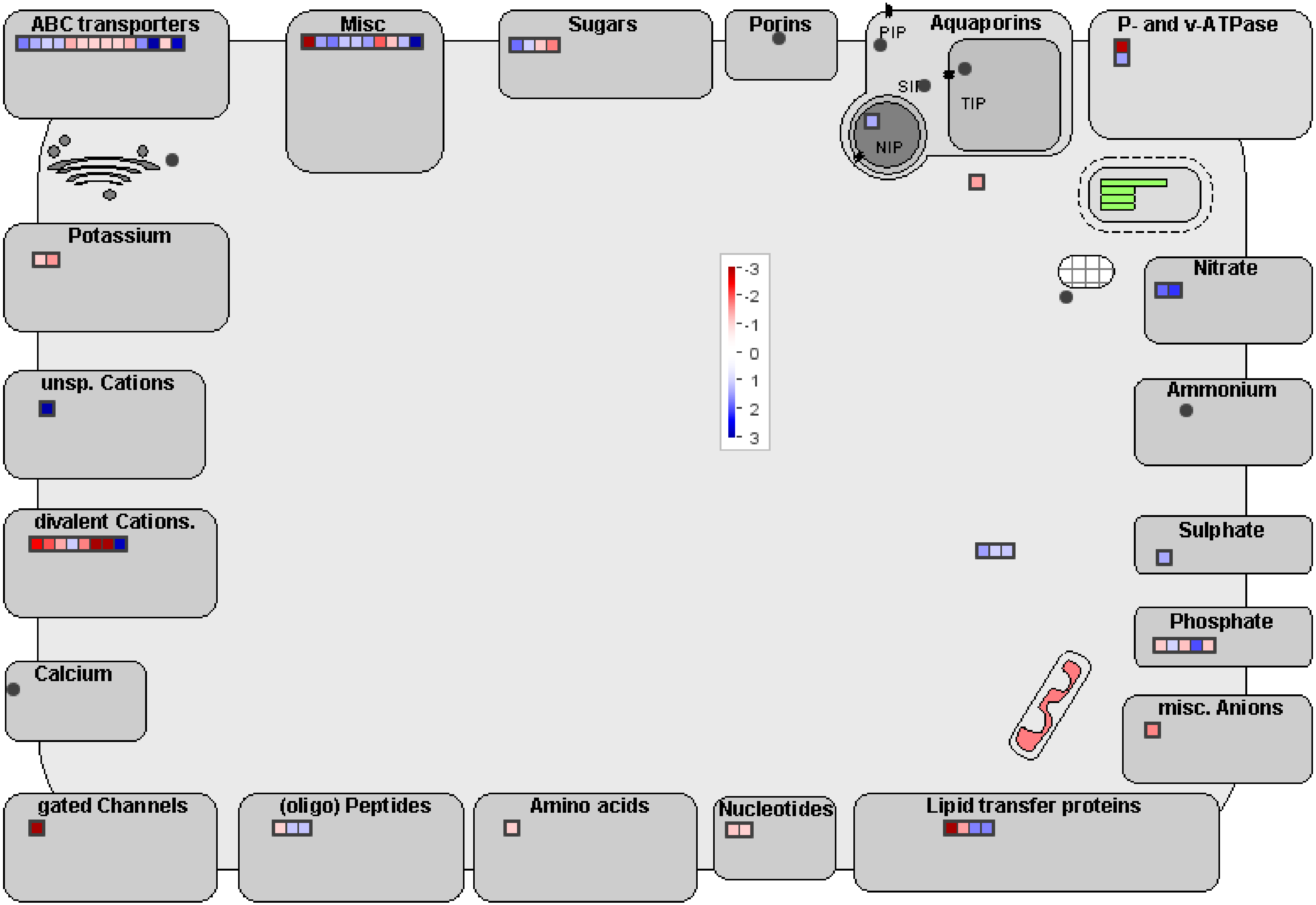

Transporter: (2) *wrky6* + *P. indica*_NP

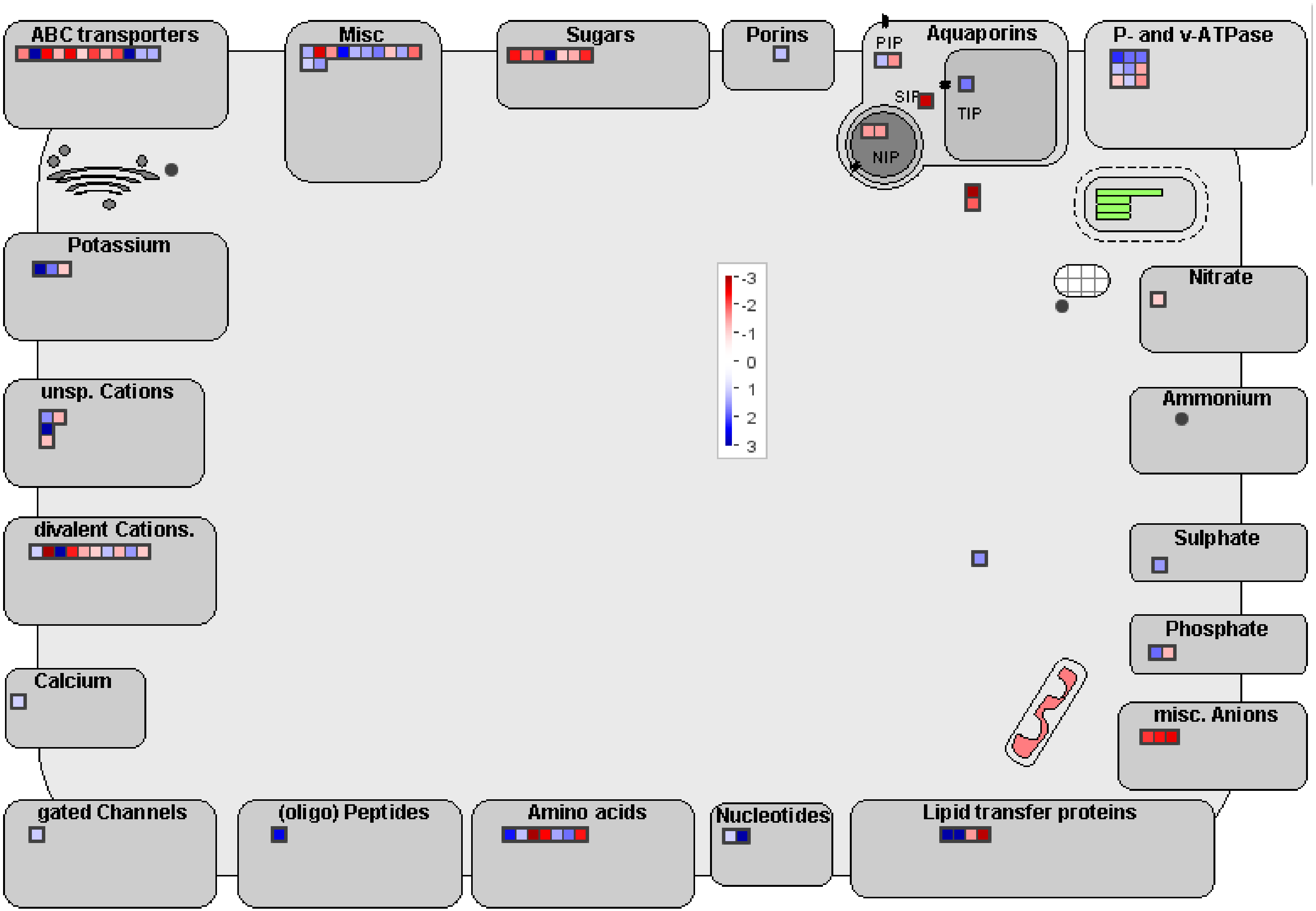

Transporter: (3) WT + *P. indica*_LP

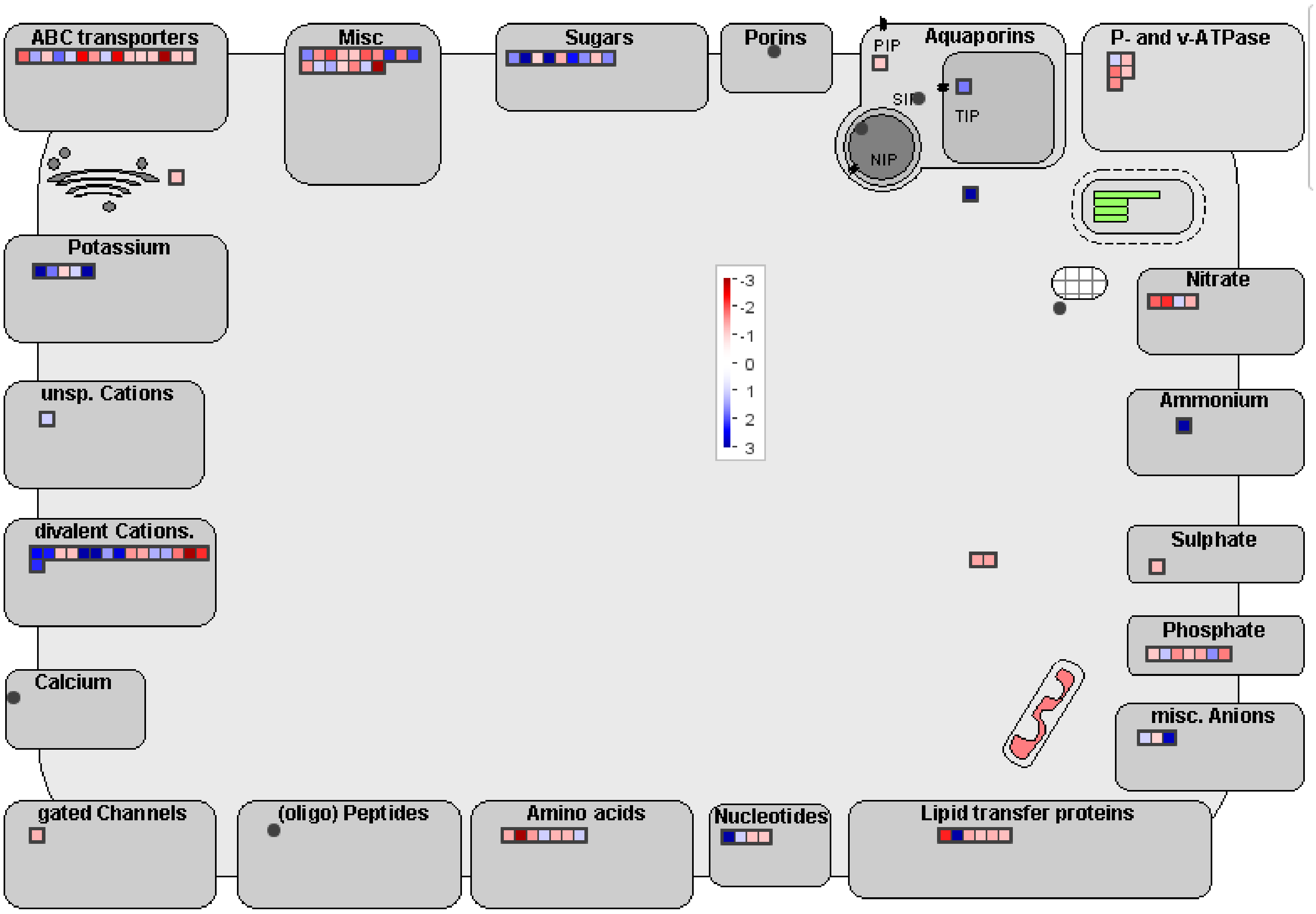

Transporter: (4) WT + *P. indica*_NP

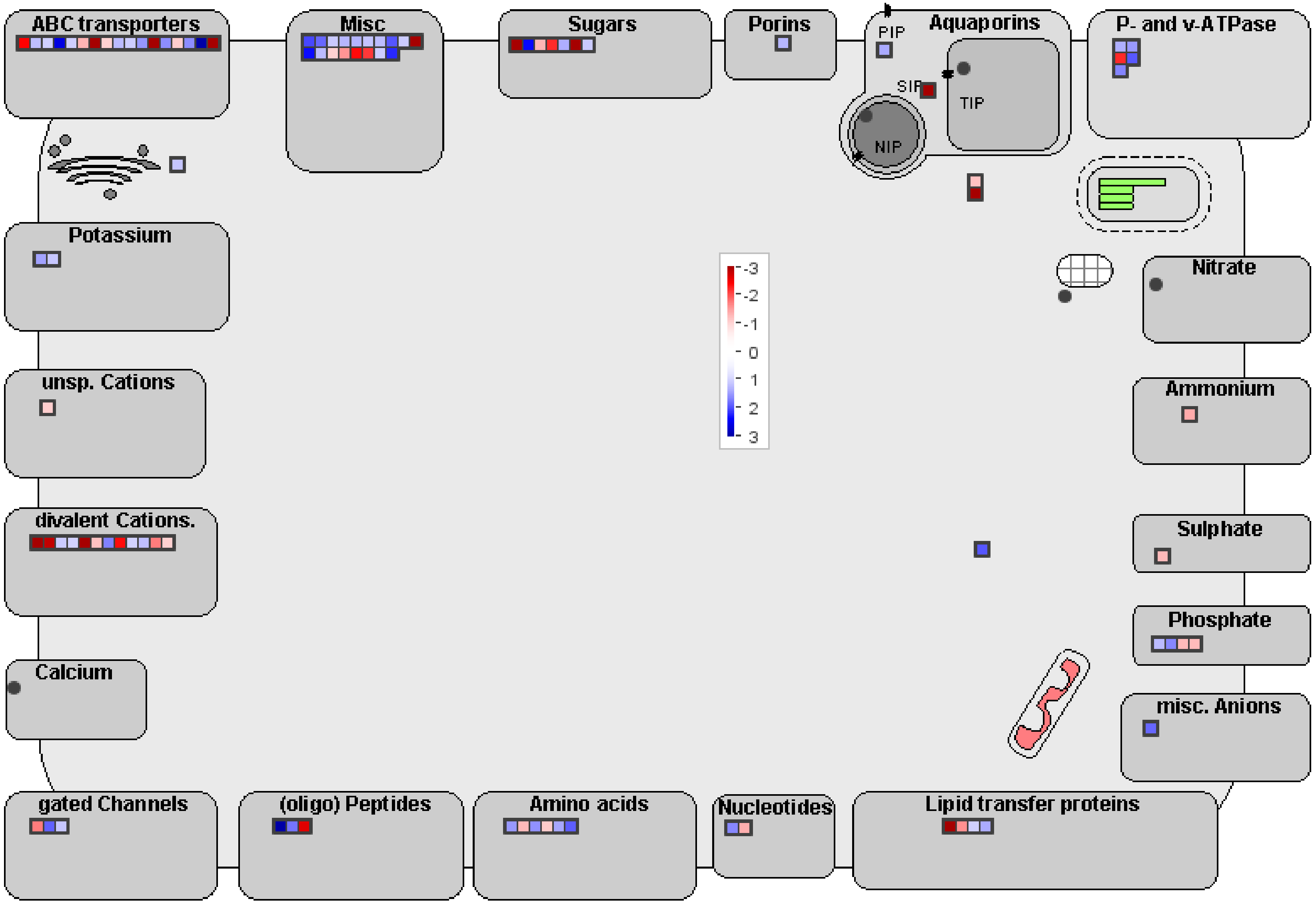

Supplementary Figure 3
(CELLULAR PROCESSES)

3-1 *wrky*6 + *P. indica*_LP
3-2 *wrky*6 + *P. indica*_NP
3-3 WT + *P. indica*_LP
3-4 WT + *P. indica*_NP

Mapman pathway depicting differentially expressed genes (2-fold) by *P. indica* in WT or *wrky6* roots grown on either NP or LP. (**Suppl. Fig. 1**) Genes coding for enzyme families, (**Suppl. Fig. 2**) for transporter families, (**Suppl. Fig. 3**) for different cellular responses and (**Suppl. Fig. 4**) for regulatory processes. Level of expression with blue is high and red is low. For more detailed information, cf. the MapMan software at http://mapman.gabipd.org/web/guest/ mapman.

Cellular processes: (1) *wrky6 + P. indica*_LP

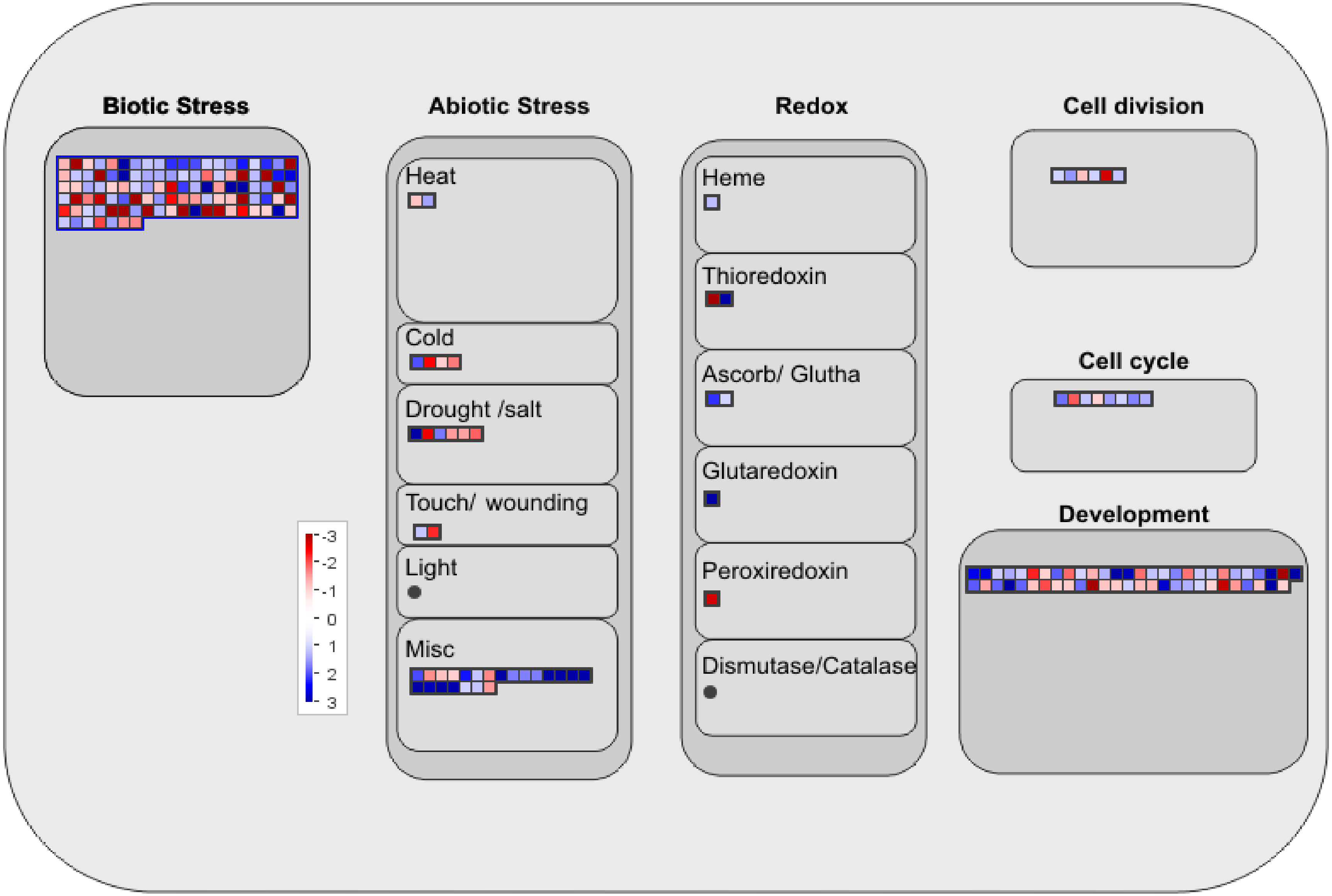

Cellular processes: (2) *wrky6 + P. indica*_NP

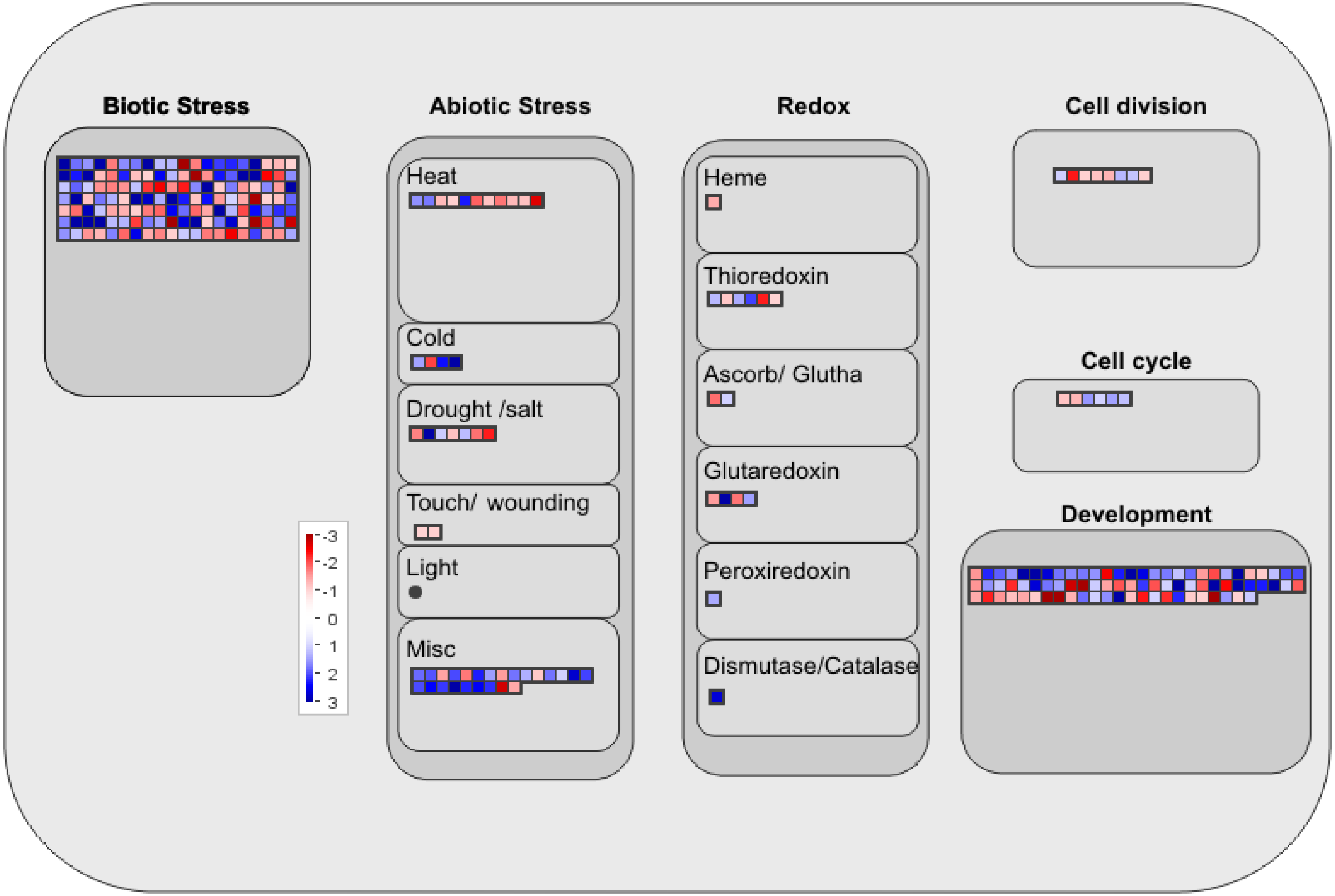

Cellular processes: (3) WT + *P. indica*_LP

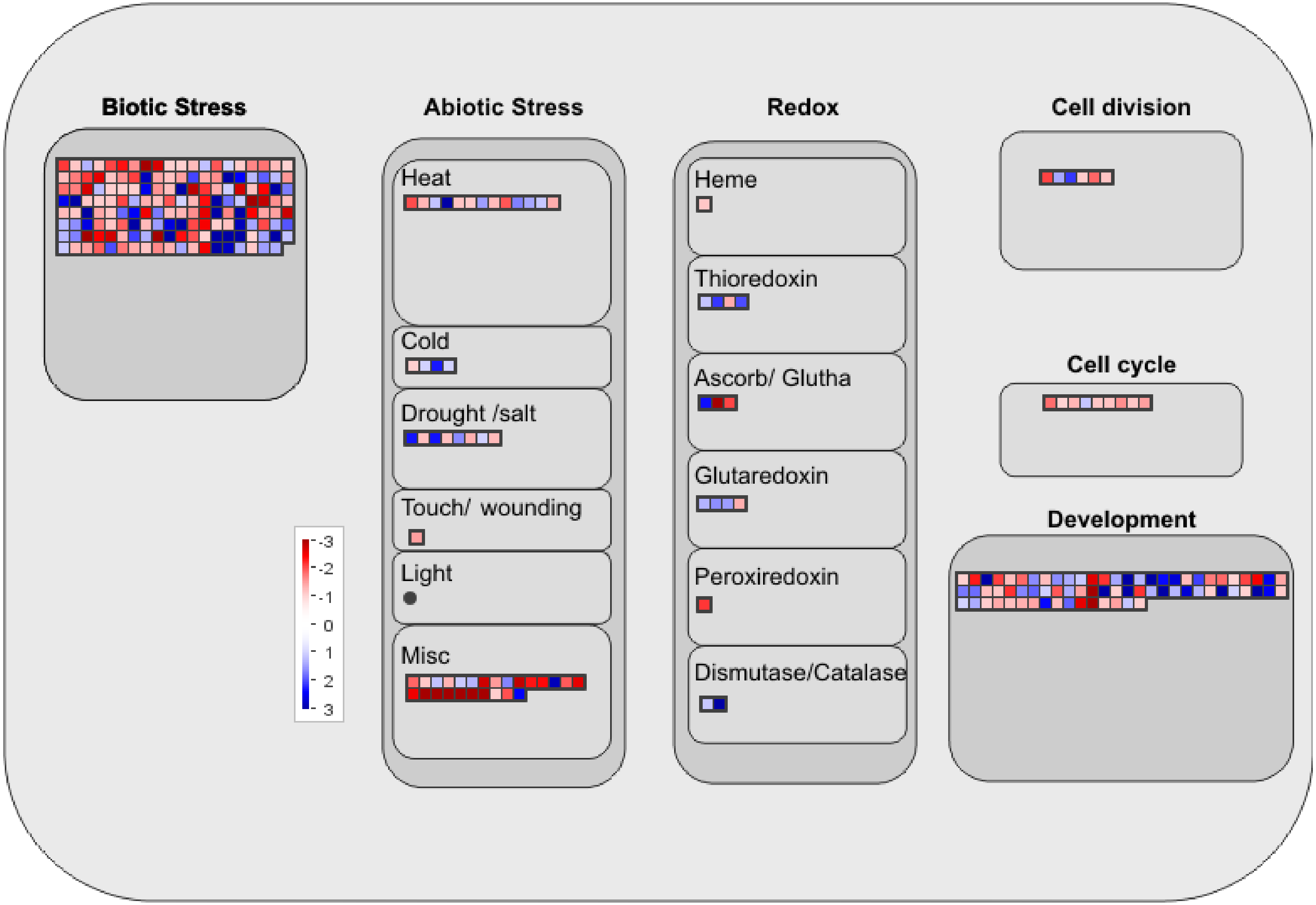

Cellular processes: (4) WT + *P. indica*_NP

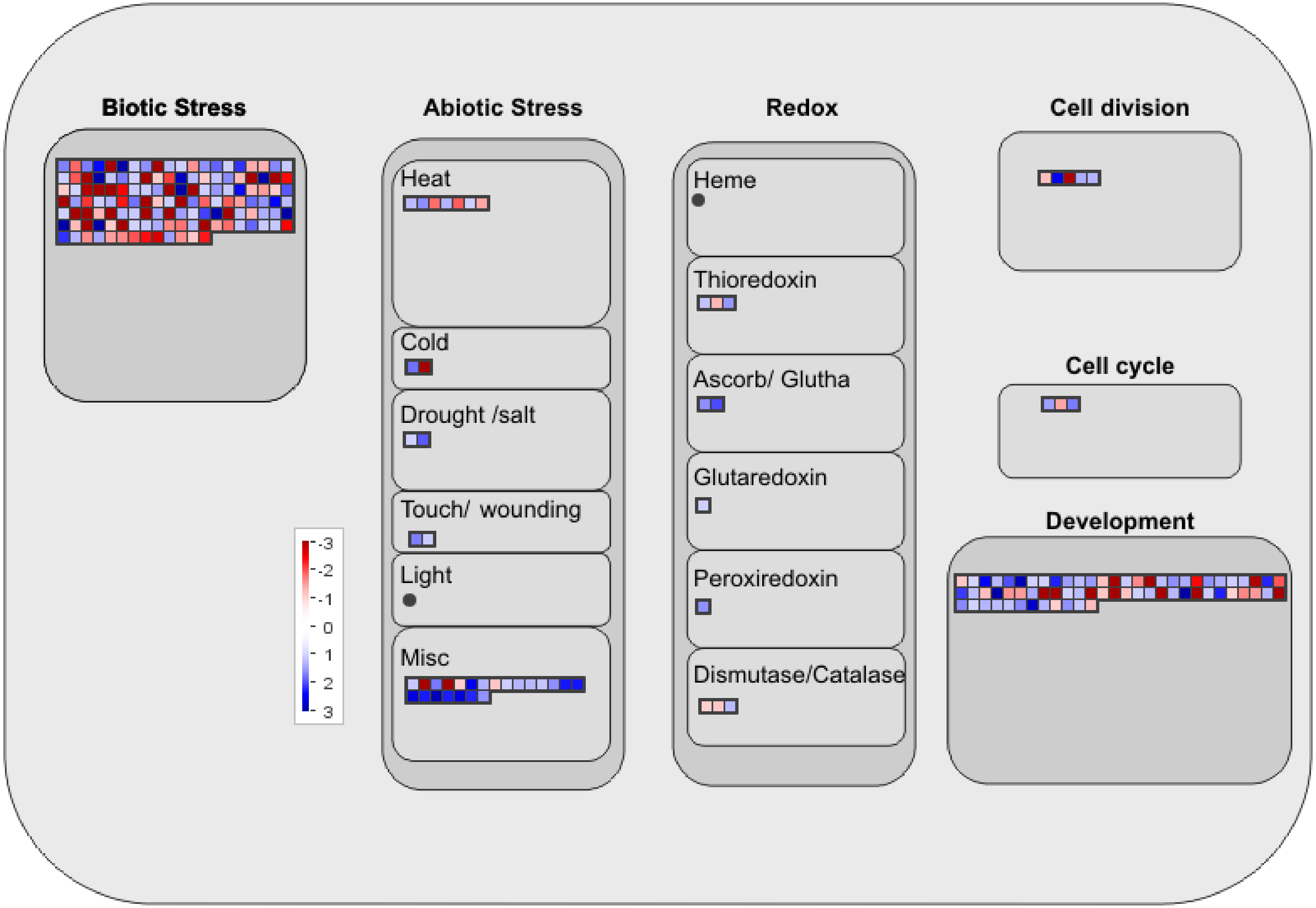

Supplementary Figure 4
(REGULATORY PROTEINS)

4-1 *wrky*6 + *P. indica*_LP
4-2 *wrky*6 + *P. indica*_NP
4-3 WT + *P. indica*_LP
4-4 WT + *P. indica*_NP

Mapman pathway depicting differentially expressed genes (2-fold) by *P. indica* in WT or *wrky6* roots grown on either NP or LP. (**Suppl. Fig. 1**) Genes coding for enzyme families, (**Suppl. Fig. 2**) for transporter families, (**Suppl. Fig. 3**) for different cellular responses and (**Suppl. Fig. 4**) for regulatory processes. Level of expression with blue is high and red is low. For more detailed information, cf. the MapMan software at http://mapman.gabipd.org/web/guest/ mapman.

Regulatory proteins: (1) *wrky6* + *P. indica*_LP

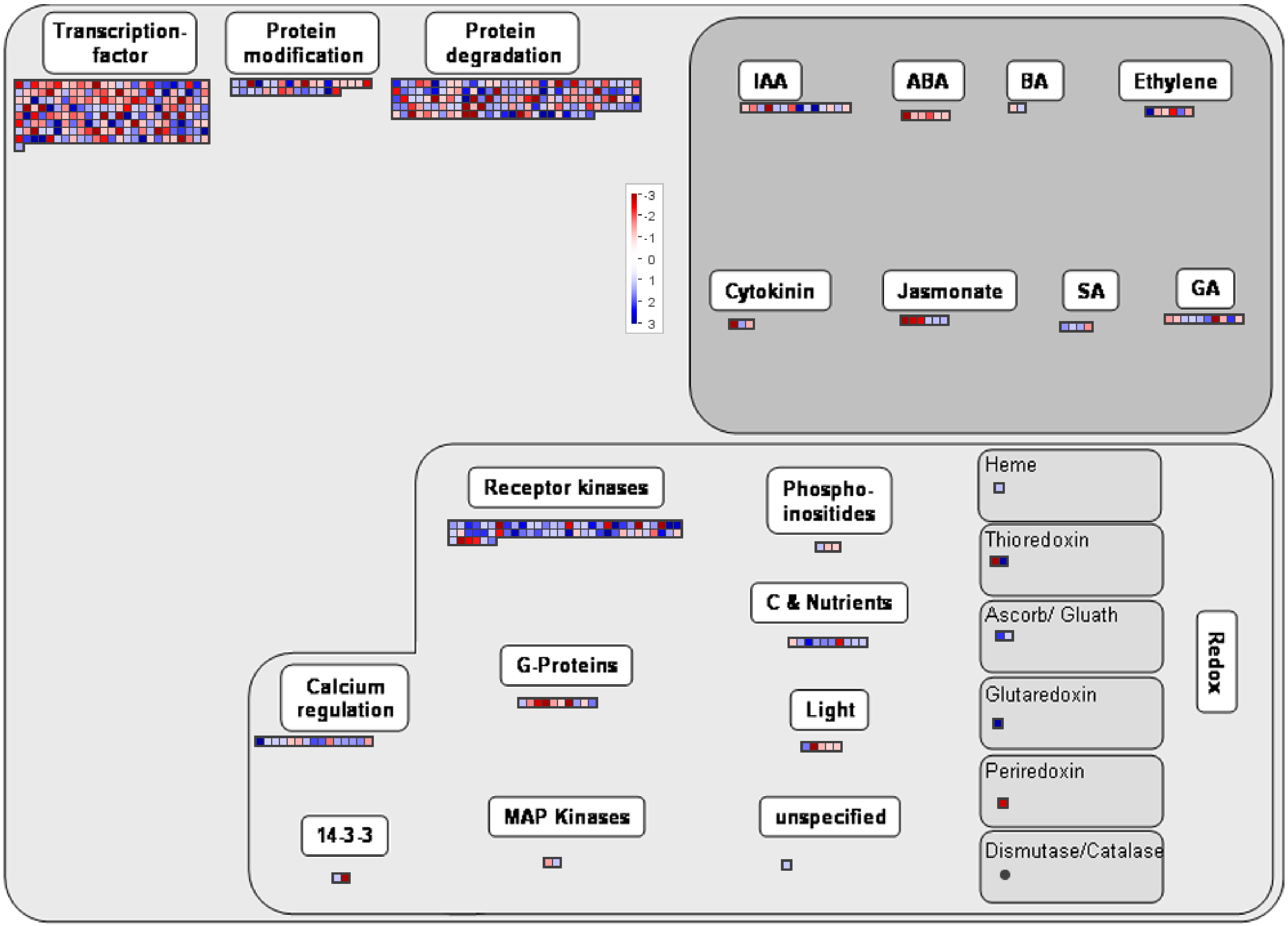

Regulatory proteins: (2) *wrky6 + P. indica*_NP

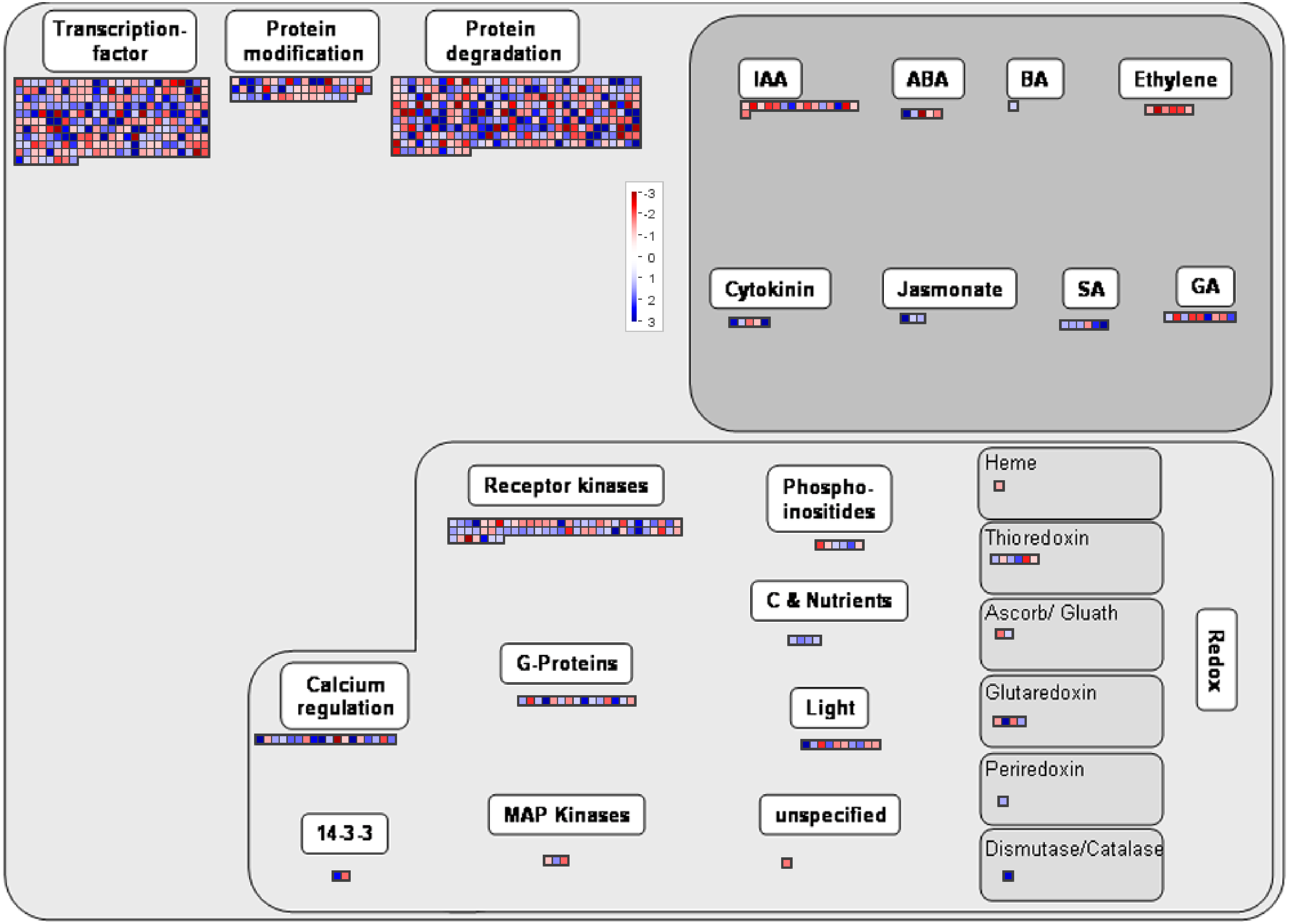

Regulatory proteins: (3) WT + *P. indica*_LP

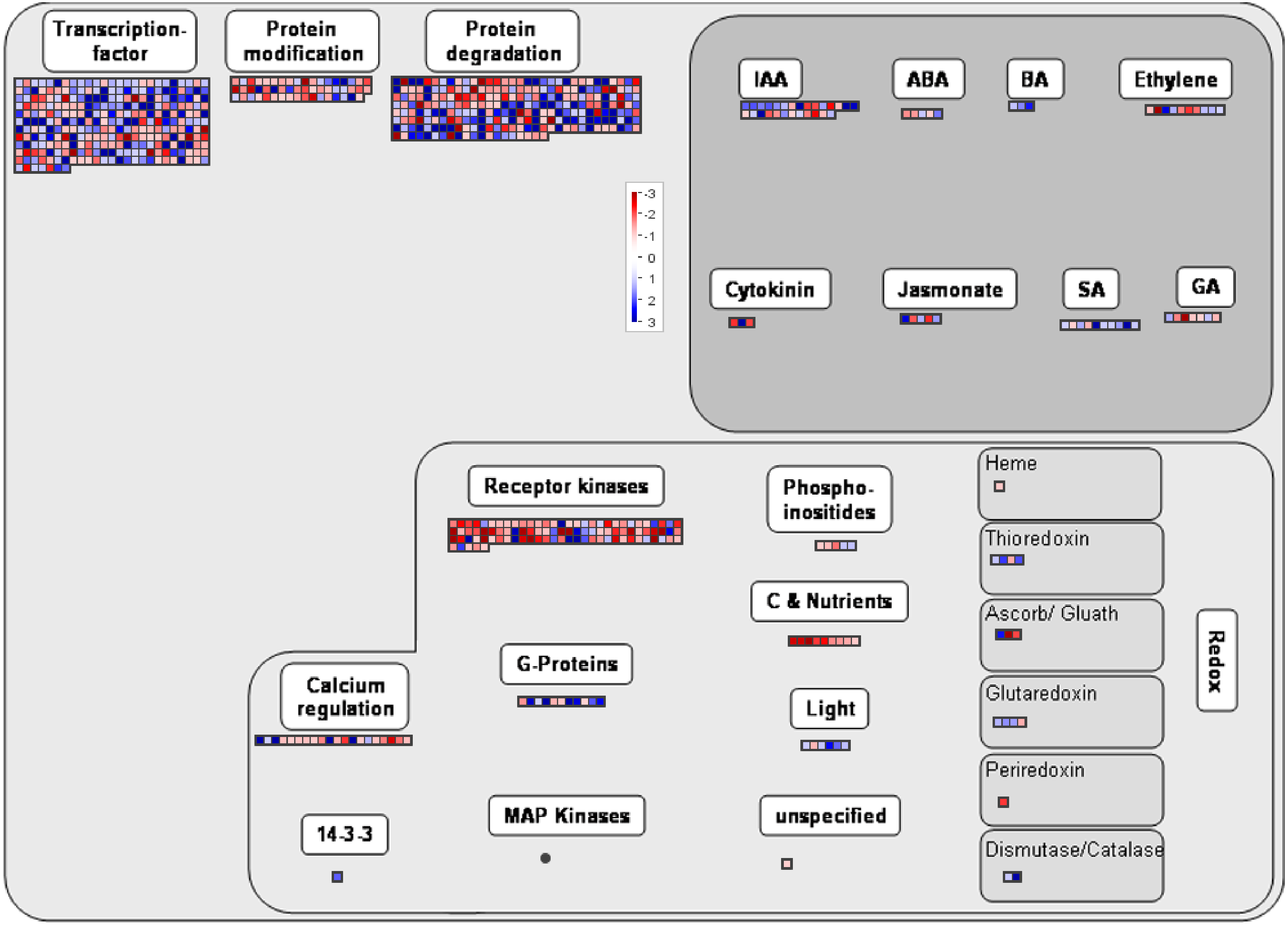

Regulatory proteins: (4) WT+ *P. indica*_NP

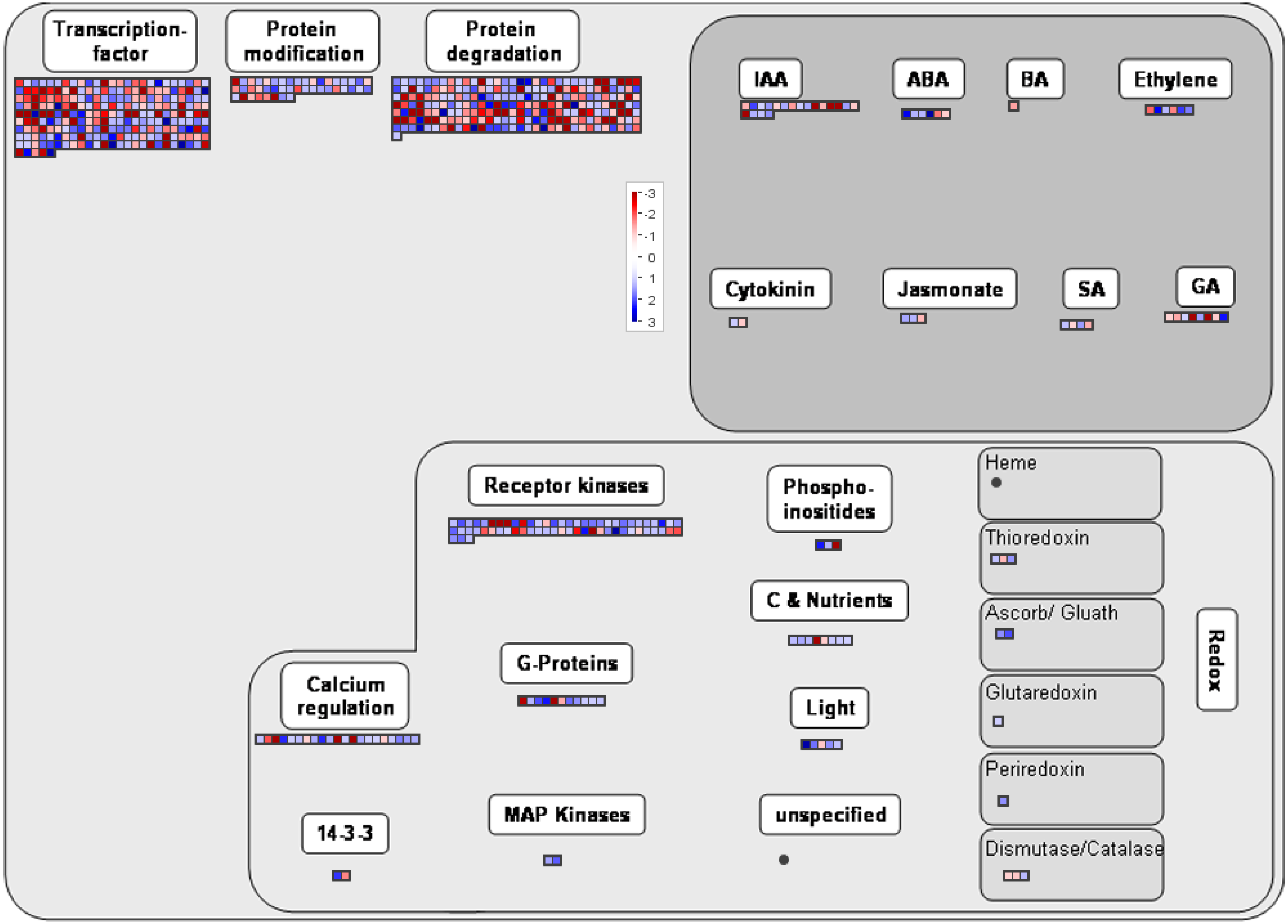

Supplement: Supplementary file 3 [file Image_1.PDF]
